# Responsive Polyelectrolyte Brushes in Applications: Functions, Stimuli, and Design Considerations

**DOI:** 10.1002/adma.202509580

**Published:** 2025-07-31

**Authors:** Leon A. Smook, Andreas Dahlin, Karin Schroën, Sissi de Beer

**Affiliations:** ^1^ Department of Molecules and Materials MESA+ Institute, University of Twente Enschede 7500 AE The Netherlands; ^2^ Department of Chemistry and Chemical Engineering Chalmers University of Technology Gothenburg 41296 Sweden; ^3^ Laboratory of Food Process Engineering Wageningen University and Research Wageningen 6708 WG The Netherlands; ^4^ Department of Membrane Science and Technology MESA+ Institute, University of Twente Enschede 7500 AE The Netherlands

**Keywords:** functional surfaces, polyelectrolyte brushes, stimulus‐response

## Abstract

Polyelectrolyte brushes are stimulus‐responsive coatings that change surface properties such as friction, adhesion, and interaction with biomolecules. Brush coatings are becoming increasingly available with improving synthesis and fabrication methods, but their use in real‐world applications is trailing behind. With their stimulus‐controlled properties, brushes can fulfill a variety of functions when they are applied in a broad spectrum of use cases ranging from tunable lubrication to ionic current rectification. In this review, summarizes the functional roles polyelectrolyte brushes can play in applications by affecting the mechanical, molecular, and electrical properties of surfaces; the main stimuli that are used to exploit the responsiveness of these coatings are discussed; and design considerations to choose an initial brush when designing systems for new applications are provided. This review concludes with a short outlook on the outstanding challenges and opportunities for applying stimulus‐responsive polyelectrolyte brushes.

## Graphical abstract

With advances in the synthesis of polyelectrolyte brushes, these coatings of surface‐grafted chains find uses in an increasing number of applications. This review summarizes three core aspects of these brushes: how they introduce functionality to surfaces; how external stimuli influence their properties; and what practical considerations should be taken into account when designing these coatings.

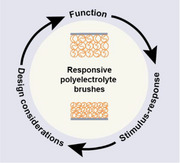

## 1 Introduction

Polyelectrolyte brushes can introduce a broad array of functionality to surfaces. One of these properties is their extremely low friction behavior,^[^ ^1^, ^2^, ^3^ ^]^ which may find applications in wear‐resistant prosthetics.^[^ ^4^ ^]^ Various other uses have also been studied and reviewed in detail over the last decade.^[^ ^5^, ^6^, ^7^ ^]^ Polyelectrolyte brushes have been used to control the adhesion of proteins at surfaces,^[^ ^8^, ^9^, ^10^, ^11^, ^12^ ^]^ increase the specificity and anti‐fouling performance of membranes,^[^ ^13^, ^14^ ^]^ enhance the functionality of batteries, fuel cells, and other electrical applications,^[^ ^15^, ^16^, ^17^ ^]^ to catalyze chemical reactions,^[^ ^18^, ^19^ ^]^ and in nanotheranostics.^[^ ^20^ ^]^ They can even be used as model systems for cellular processes^[^ ^21^ ^]^ or perform a size‐selective adsorption of gold nanoparticles.^[^ ^22^, ^23^ ^]^

Polyelectrolyte brushes change the properties of the surface they are attached to. The polymers in these coatings are grafted to the substrate. When individual chains are grafted to substrates, they form mushroom‐like structures, but if these chains are grafted at a high density, the mushroom structures overlap. This confines the space for each polymer chain and forces the chains to stretch in the direction away from the grafting plane to form a brush.^[^ ^24^, ^25^, ^26^ ^]^ A key design parameter of polyelectrolyte brushes is the grafting density.^[^ ^27^, ^28^, ^29^ ^]^ This parameter determines the degree of confinement of the grafted chains and significantly influences physicochemical behavior. Besides the grafting density, polyelectrolyte brushes can be described based on the properties of the grafted polymers, including their molecular weight,^[^ ^7^ ^]^ chain length dispersity,^[^ ^30^, ^31^, ^32^, ^33^ ^]^ and macromolecular connectivity and design.^[^ ^34^, ^35^, ^36^, ^37^ ^]^ Combined, these parameters provide a rich space throughout which the properties of polyelectrolyte brushes can be tuned.

It is no trivial task to attach polymer chains to a surface at high grafting densities^[^ ^38^, ^39^, ^40^, ^41^ ^]^ or to grow them from a surface in a uniform and controlled manner,^[^ ^5^ ^]^ especially if the polymers are charged and experience electric repulsion. The use of polyelectrolyte brushes in real‐world applications has found only limited traction because the synthesis of polyelectrolyte brushes has long been reserved to experts. Nevertheless, developments in polymer brush synthesis^[^ ^7^, ^42^, ^43^, ^44^, ^45^ ^]^ decreased the level of expertise required to prepare polymer brushes. Now, anyone with a chemical lab, chemical skills, and the right chemicals can grow brushes and with experience, even control their design characteristics.

The properties of polyelectrolyte brushes result from the brush design in conjunction with the physical environment. Relevant properties of brushes include the brush thickness,^[^ ^46^, ^47^, ^48^ ^]^ friction coefficient,^[^ ^1^, ^49^, ^50^, ^51^ ^]^ and wettability.^[^ ^52^, ^53^ ^]^ Since these properties depend on both the brush design and the external physical environment, it is possible to modulate brush properties through changes in the environment. Such stimulus‐responsive systems can then find uses in a variety of applications.^[^ ^20^, ^54^, ^55^, ^56^ ^]^

The design‐property relationship in polyelectrolyte brushes has been the subject of a large body of work in the literature.^[^ ^16^, ^52^, ^55^, ^57^, ^58^, ^59^, ^60^, ^61^ ^]^ Much of this (theoretical) work aims to describe the thermodynamic equilibrium structure of the brushes via techniques ranging from scaling analyses^[^ ^2^, ^62^, ^63^, ^64^ ^]^ and self‐consistent field theories^[^ ^61^, ^65^ ^]^ to coarse‐grained^[^ ^27^, ^66^, ^67^ ^]^ and atomistic^[^ ^68^, ^69^, ^70^, ^71^ ^]^ molecular dynamics simulations. The equilibrium structure of polyelectrolyte brushes results from a delicate balance between various effects.^[^ ^72^, ^73^ ^]^ These effects include the entropic elasticity of the grafted chains; the osmotic pressure of confined counterions in the brush; excluded volume interactions between chain segments; charge correlations between ions; dispersive interactions between ions, monomers, and solvent; the chemical nature of counterions; and (induced) dipole interactions. As a result, accurate prediction of equilibrium brush properties based on design parameters is rather challenging. However, by combining experimental results and numerical approaches with increasing computational power, we can study these system over a larger range of time and length‐scales to improve our understanding of complexity in these systems.

Several excellent reviews have been written on polyelectrolyte brushes before, ranging from the classics summarizing the initial research on these systems.^[^ ^74^, ^75^ ^]^ to more recent reviews with a narrower focus on specific types of polyelectrolyte brushes^[^ ^76^, ^77^, ^78^, ^79^ ^]^ or research method.^[^ ^55^, ^80^, ^81^, ^82^, ^83^ ^]^ In this review, we provide an overview of strong and weak polyelectrolyte brushes with a special emphasis on their stimulus‐responsive character as well as insights for researchers that want to use stimulus‐responsive brushes in their applications. Our discussion is limited to brushes that contain either only positively charged moieties or only negatively charged ones. We highlight the practical functions that polyelectrolyte brushes can play in applications, using insights from experimental and theoretical studies.

**Figure 1:**
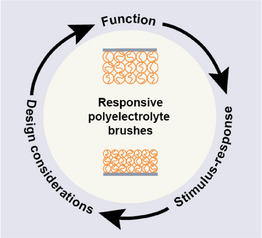
Overview of the contents. The review first presents how responsive polyelectrolyte brushes can be used in applications to create functionality (Section 2), followed by a discussion how response can be achieved via stimuli (Section 3), and finally a discussion on the implementation of these insights into the design of a polyelectrolyte brush (Section 4).

The review is constructed of three different parts as illustrated in **Figure** **1**. In Section 2, we summarize functions that stimulus‐responsive polyelectrolyte brushes can have in applications. This section aims to inspire the reader to consider the breadth of applications where brushes can find a use. In Section 3, we describe the most important stimuli that can be employed to change the properties of polymer brushes such as their height, friction behavior, and permeability. This section aims to summarize both how these stimuli create a desired response and simultaneously provides an overview of factors to control or prevent undesired responses. In Section 4, we provide a primer for the synthesis and chemistry of polyelectrolyte brushes as well as an overview of commonly‐used monomers. This section is intended as a starting point for the design and synthesis of a polyelectrolyte brush for an application. Finally, in Section 5, we conclude with an outlook to highlight important aspects related to current developments in science and society.

## 2 Functions of Polyelectrolyte Brushes

**Figure 2:**
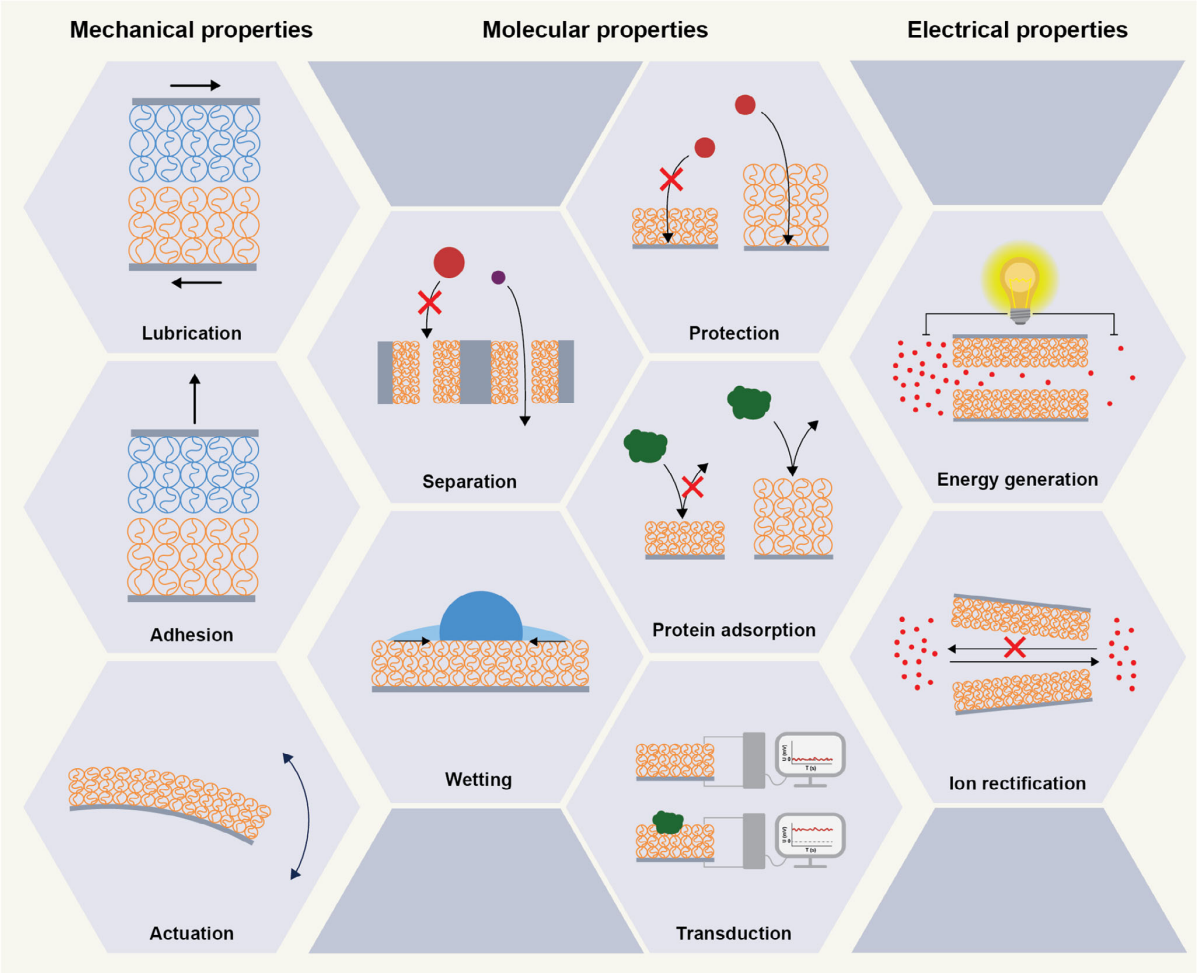
Stimulus‐responsive polyelectrolyte brushes has been used in a variety of applications exploiting changes in various brush properties. Their response is used to influence the mechanical properties of the surface (friction–2.1.1, adhesion–2.1.2, stress‐induced actuation–2.1.3); to modify the interaction with molecules (molecular barriers–2.2.1, membranes–2.2.2, anti‐fouling coatings–2.2.3, wettability‐control–2.2.4, and molecular transduction–2.2.5); and to affect the transport of charged species for electrical applications (energy recovery–2.3.1, ionic diodes–2.3.2).

The function of brushes in applications can be sorted into three categories as illustrated in **Figure** **2**. First, brushes can modify the mechanical properties of the system: They can tune properties such as adhesion and friction and they act as soft actuators (Section 2.1). Second, brushes can modify how the system interacts with molecules in the surrounding medium (Section 2.2). Examples of this category include reversible barriers, membrane functionalization, and controlled protein adsorption and desorption. Finally, brushes can modulate the electric properties of systems by affecting ionic transport: They can be used in energy conversion and as a key element in ionic diodes (Section 2.3).

### 2.1 Manipulating Mechanical Properties

One of the first remarkable properties of polyelectrolyte brushes that was observed is their low friction.^[^ ^49^, ^50^, ^56^ ^]^ It was already known that brushes reduce friction between surfaces,^[^ ^84^ ^]^ but (like‐charged) polyelectrolyte brushes do this even better (Section 2.1.1). Similarly, brushes affect the adhesion between interfaces (Section 2.1.2). As a result of grafting, brushes exert stresses on the substrates they are grafted to. These stresses can be harnessed in the form of mechanical actuation (Section 2.1.3)

The mechanical properties of brushes are intrinsically related to their behavior under compression. Typically, the pressure exerted by a compressed system of polymer brushes stems from an interpenetration and a compression contribution, as was reviewed in detail by Kreer.^[^ ^1^ ^]^ In polyelectrolyte brushes, the compressive force is dominated by steric repulsion between the polymer segments in the opposing brushes. However, it is additionally strongly affected by electrostatic effects since counterions contribute to the osmotic pressure, so the compressive force can vary when salt is varied and the brush transitions between the osmotic and salted regime.^[^ ^85^ ^]^ (For a description of these regimes, see Section 3.1.1)

#### 2.1.1 Lubrication

Polyelectrolyte brushes can efficiently reduce friction between surfaces.^[^ ^49^, ^50^, ^56^, ^86^, ^87^, ^88^, ^89^, ^90^, ^91^, ^92^, ^93^ ^]^ In water, the friction coefficient can be as low as 0.001.^[^ ^2^, ^94^ ^]^ This friction reduction is attributed to the combined effect of the lack of interpenetration between opposing brushes and the hydration layer around the charges on the polyelectrolyte.^[^ ^2^, ^64^ ^]^ With this understanding, one can try to tune the friction coefficient between two opposing brushes. For instance, the friction between poly(methacrylic acid) (PMAA)‐coated particles in a rotating drum experiment can be influenced significantly by changing the pH.^[^ ^95^ ^]^

On the one hand, one can affect the friction through the interpenetration between opposing brushes. For instance, one can create a macroscopic interpenetration between two surfaces by depositing brush‐coated nanoparticles on the substrate. If the spacing between the particles is large enough, the particles will interpenetrate and increase the friction.^[^ ^97^ ^]^ Alternatively, the interpenetration can also be tuned through external conditions such as pH. Abbott et al. studied the interpenetration of a polybasic PDMAEMA (poly[2‐(dimethylamino)ethyl methacrylate]) brush and a PEO (poly(ethylene oxide)) brush and found that the degree of interpenetration strongly increases when the pH is reduced from 10 to 5 or lower because the neutral polymer acts as a diluent for the highly charged brush.^[^ ^101^ ^]^ In another study, Raftari et al. studied the friction and adhesion between PDMAEMA brush substrates with polybasic PDMAEMA and a polyacidic PMAA (poly(methacrylic acid) coated cantilever tip.^[^ ^98^ ^]^ The friction between the substrate and a PDMAEMA tip gradually increases with increasing pH (and thus decreasing charge on the polymer). The friction between the substrate and acid PMAA tip went through a maximum around pH 6, probably due to acid‐base interactions.

On the other hand, one can affect the friction through the hydration layer around the grafted polyelectrolyte chains. This hydration layer is most directly influenced by the counterions in the brush. Yu et al.^[^ ^51^ ^]^ studied the friction between PSS (poly(styrene sulphonate)) brushes and showed that even minute concentrations of multivalent ions like Y^3+^, Ca^2+^, Ba^2+^ can cause electrostatic bridging which drastically increases the friction force between brush layers. Alternatively, one can affect the brush friction through the introduction of charged surfactants^[^ ^87^ ^]^ or hydrophobic counterions.^[^ ^86^ ^]^

To conclude, many stimuli can be used to change the friction with a polyelectrolyte brush by affecting interpenetration and hydration properties of brushes. We summarize these stimuli and their effect on friction in **Table** **1**.

**Table 1:** Tunable lubrication with polyelectrolyte brushes.

Stimulus	Brush	Countersurface	Switch	Switch factor^a)^	Refs.
salt concentration	PSS	PSS	Na^+^, Y^3+^, Ca^2+^, Ba^2+^	1000	^[^ ^51^ ^]^
	PSPMA	PDMS	K^+^, Cu^2+^, Fe^3+^	5	^[^ ^86^ ^]^
	UHMWPE‐PDMAEMA	Si_3_N_4_	NaCl: 0 to 6.1 M	< 2	^[^ ^96^ ^]^
	UHMWPE‐PSPMA	Si_3_N_4_	NaCl: 0 to 6.1 M	ca. 2	^[^ ^96^ ^]^
counterion exchange	PMETAC	PDMS	Cl^‐^, ClO_4_^‐^, PF_6_^‐^, TFSI^‐^	1000	^[^ ^86^ ^]^
	PSPMA	PDMS	K^+^, TBAB, DTAB, CTAB	100	^[^ ^86^ ^]^
	PMAA	PDMS	Na^+^, NH_4_^+^, TMAB, TEAB, TBAB	20	^[^ ^86^ ^]^
	UHMWPE‐PSPMA	Si_3_N_4_	Na^+^, K^+^, Mg^2+^, Ca^2+^, La^3+^, Fe^3+^	ca. 2	^[^ ^96^ ^]^
	UHMWPE‐PDMAEMA	Si_3_N_4_	Na^+^, K^+^, Mg^2+^, Ca^2+^, La^3+^, Fe^3+^	ca. 1	^[^ ^96^ ^]^
	UHMWPE‐PSPMA	Si_3_N_4_	Cl^‐^, Br^‐^, NO_3_^‐^, SO_4_^2‐^, PF_6_^‐^, TFSI^‐^	1	^[^ ^96^ ^]^
	UHMWPE‐PDMAEMA	Si_3_N_4_	Cl^‐^, Br^‐^, NO_3_^‐^, SO_4_^2‐^, PF_6_^‐^, TFSI^‐^	ca. 2	^[^ ^96^ ^]^
surfactant concentration	PMETAC	PDMS	SDS	150	^[^ ^87^ ^]^
	PSPMA	PDMS	CTAB	30	^[^ ^87^ ^]^
pH	PMAA	PDMS	pH: 2 to 12	500	^[^ ^86^ ^]^
	PDMAEMA	gold	pH: 3 to 11	10	^[^ ^88^ ^]^
	P2VP	Si / Si_3_N_4_ / PS / PAA	pH: 2 / ethanol	ca. 20	^[^ ^89^ ^]^
	SiO_2_‐*g*‐PDMAEMA	silica / SiO_2_‐*g*‐PDMAEMA	pH: 5 to 9	50	^[^ ^97^ ^]^
	PDMAEMA	PDMAEMA / PMAA	pH: 1 to 12	20	^[^ ^98^ ^]^
	PDMAEMA	Si_3_N_4_	pH: 1 to 8	12	^[^ ^90^ ^]^
	P(DMAEMA‐*b*‐MAzo)	PDMS	pH: 4 to 10	ca. 2	^[^ ^99^ ^]^
humidity	PSPMA	PDMS	RH: 0% to 95%	100	^[^ ^49^ ^]^
	PMETAC	PDMS	RH: 0% to 95%	100	^[^ ^49^ ^]^
electric field	PAHPS	PAHPS	± 0.4V	<2	^[^ ^100^ ^]^
light	P(DMAEMA‐*b*‐MAzo)	PDMS	UV‐Vis	<2	^[^ ^99^ ^]^

^a)^ Indication of the ratio between the friction coefficient in the highest friction state and the lowest friction state.

#### 2.1.2 Adhesion

Adhesion between brush‐coated surfaces is often discussed in conjunction with friction.^[^ ^97^, ^98^, ^102^, ^103^, ^104^, ^105^, ^106^, ^107^ ^]^ Different effects can contribute to adhesion, including dispersive interactions, charged interactions, and ionic bridging. When surfaces are oppositely charged, this leads to adhesion between the surfaces. This adhesion can bond with lap shear strengths of over 1 MPa^[^ ^106^ ^]^ (which is stronger than duct tape). Similarly, silica cubes could be made to self‐assemble when one was functionalized with poly[(2‐methacryloyloxy)ethyl trimethylammonium chloride] (PMETAC) brushes and the other with poly(3‐sulfopropyl methacrylate) (PSPMA) brushes.^[^ ^108^ ^]^ In both systems, the adhesion was reversible in salt solutions.^[^ ^106^, ^108^ ^]^ To achieve adhesion between the surfaces, it is crucial that brushes are sufficiently thick.^[^ ^108^, ^109^ ^]^

**Figure 3:**
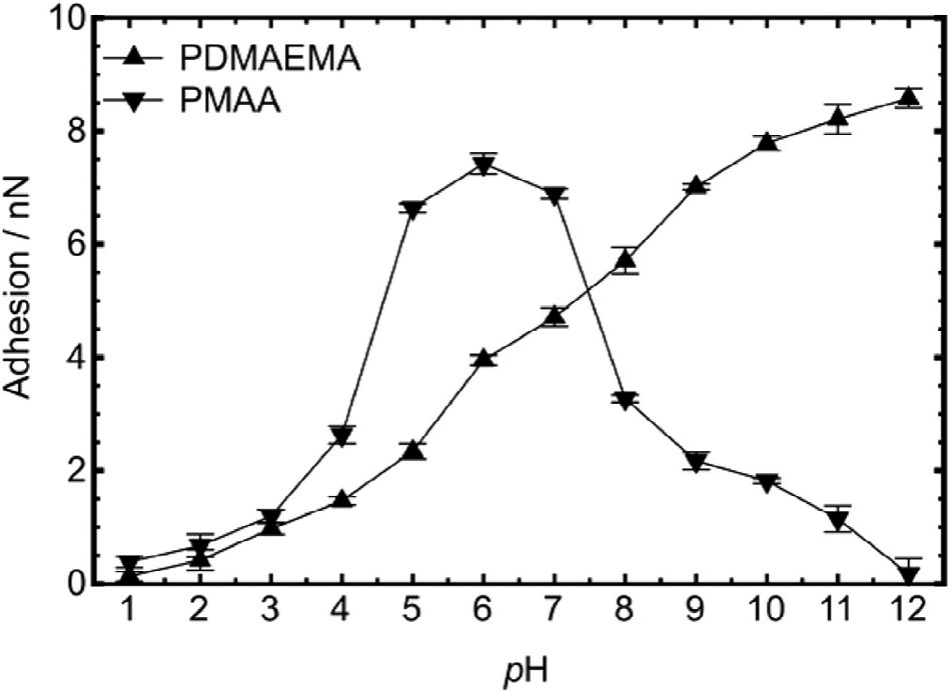
Adhesion between PDMAEMA‐ and PMAA‐coated AFM tips (radius = 20 nm) with a planar PDMAEMA brush as a function of pH. Reproduced under terms of the CC‐BY license.^[^ ^98^ ^]^ Copyright 2015, The Authors, published by American Chemical Society.

When the surfaces consist of weak polyelectrolyte brushes, changes in pH will affect the charge of the polymer, creating an additional mechanism to tune adhesion. For instance, the interaction strength between opposing PMAA and PDMAEMA brushes has a maximum around pH 6 where both brushes are partially charged as illustrated in **Figure** **3**.^[^ ^98^ ^]^ Similar adhesion effects can be observed when one surface consists of a gel.^[^ ^109^, ^110^, ^111^ ^]^

For similarly charged brushes (PDMAEMA on PDMAEMA), the adhesion steadily increases with pH where the brushes are more ionized and better solvated.^[^ ^98^ ^]^ This is somewhat surprising and cannot be explained based on hydrogen bonding. Nevertheless, the authors note that this effect coincides with adhesion over a longer distance as indicated by a maximum pull‐off force at a larger displacement. Other external factors can change these adhesion properties between similarly charged brushes as well. For instance, the addition of multivalent calcium ions to a system of opposing PAA (poly(acrylic acid)) brushes can lead to bridging interactions between opposing brushes at concentrations above approximately 1 mM.^[^ ^112^, ^113^ ^]^ The formation of calcium bridges also depends on pH: at low pH values when the PAA is mostly neutral, adhesion between opposing surfaces is minimal.^[^ ^112^ ^]^ Similarly, adhesion between opposing brushes can be induced by trivalent ions.^[^ ^105^, ^114^ ^]^ Along with other effects, such bridging interactions can contribute to aggregation of brush‐stabilized nanoparticles in solutions of divalent ions.^[^ ^113^ ^]^

#### 2.1.3 Mechanical Actuation

When polyelectrolyte chains are grafted to substrates, they are closely packed laterally compared to their equivalent bulk configuration. As a result, the brush exerts a compressive stress on the surface they are attached to^[^ ^115^ ^]^ and this stress may be exploited if the substrate is flexible.^[^ ^116^ ^]^ Therefore, if one can influence the compressive stresses in the brush, one may transduce this to mechanical motion and create an actuator.

In liquids, polyelectrolyte brushes confine their counterions to the volume they occupy in low‐salt media and this confinement introduces a stress in the brush. Zhou et al. exploited this effect by grafting PMEP (poly(methacryloyl ethylene phosphate)) brushes to cantilevers and observed that the cantilever had different degrees of bending depending on external conditions such as pH and salt concentration.^[^ ^117^ ^]^ Later, Zhou et al. extended this idea by grafting a PMETAC (poly[(2‐methacryloyloxy)ethyl trimethylammonium chloride]) brush to a gold‐coated cantilever.^[^ ^118^ ^]^ In this setup, the ions in the solution could be attracted or repelled from the electrode with a bias potential, leading to varying ion concentrations in the brush, changing osmotic pressures, and finally, electrically actuated bending of the cantilever.

In air, polyelectrolyte brushes can absorb vapor and swell as a result.^[^ ^46^, ^119^, ^120^ ^]^ This swelling introduces additional tension on the substrate that can be exploited. Li et al. created a PDMS (polydimethylsiloxane) substrate coated with a PSPMA brush.^[^ ^121^ ^]^ When exposed to water vapor, this substrate showed significant bending at macroscopic scales such that a self‐walking object was created.

### 2.2 Modifying Interactions Between System and Molecules

When polyelectrolyte brushes are affected by external triggers, their interaction with molecules in the surrounding medium can alter as a result of structural or chemical changes. First, a brush can swell or collapse based on the trigger. As a result, both the height and the average polymer density in the brush layer change. These changes can affect how molecules are transported through the brushes toward the grafting surface (Section 2.2.1) and past the brushes into a porous support (Section 2.2.2). Second, external changes can affect the interaction between the grafted polyelectrolyte and components in the surrounding solution. For instance, proteins readily adsorb in some conditions while they readily desorb in others as discussed in Section 2.2.3. Third, brushes affect the interaction of surfaces with liquids, changing the wettability of the substrate (Section 2.2.4) Finally, the properties of brushes can change through the interaction with specific molecules. These effects allow brushes to be used as molecular transducers in sensors (Section 2.2.5).

#### 2.2.1 Protection: Molecular Barriers Through Controlled Brush Response

Polyelectrolyte brushes can form a reversible barrier at catalytic surfaces. Many reaction are catalyzed by solids, so a key aspect of these reactions is the accessibility of the surfaces. Brushes can act as reversible barriers and thus allow one to control the rate of diffusion and reaction.^[^ ^122^, ^123^, ^124^ ^]^ A striking example of this principle is the modulation of the speed of self‐propelled micro‐/nanomotors.^[^ ^125^ ^]^ Such micro‐/nanomotors are Janus particles where one half–the engine–catalyzes a chemical reaction and the other half is inert, leading to a propulsion mechanism depending on the rate of reaction. The engines of these nanoparticles were coated with a PMETAC brush of approximately 60 nm thick, which allows for control over the access of reactants to the surface. The rate of reaction could be tuned by varying density of the PMETAC brush with various chaotropic and kosmotropic counterions, and the particle speed could be continuously varied.

A similar barrier effect has been achieved on other electrochemical surfaces. For instance, PMETAC brushes have been grafted on a conducting polyaminobenzylamine substrate.^[^ ^126^ ^]^ The PMETAC brush could modulate the electrochemical response of the substrate via ion‐induced collapse (see also Section 3.1.1). To illustrate, in a 50 mM NaClO_4_ solution the brush collapsed and the redox transformation of the substrate could be suppressed, while in a 50 mM NaCl solution, the transformation readily proceeded with a brush under swollen conditions. In fact, this effect was demonstrated to be reversible upon exchanging the NaClO_4_ and NaCl solutions. In another work, poly(4‐vinylpyridine) brushes were grafted to ITO electrodes.^[^ ^127^ ^]^ These brushes can undergo a pH‐driven collapse which allows for control over the reduction and oxidation of [Fe(CN)O_6_]^3‐^ at the electrode. The response characteristics of this system showed memristic behavior.^[^ ^127^ ^]^ The same authors later hypothesized that these effects may find applications in biocomputing systems.^[^ ^128^ ^]^

Polyelectrolyte brushes that restrict access to electrode surfaces could both provide control over the electrode reactions and protection against biological fouling.^[^ ^129^ ^]^ However, many questions remain. For instance, it is reasonable to assume that the mobility of reactants inside a brush is a key factor in these applications. In fact, recent atomistic simulations of dense polyelectrolyte brushes with grafting densities around 0.2 chains/nm^[^ ^69^, ^130^, ^131^ ^]^ indicate that the mobility of water, counterions, and small molecules are reduced in brush environments, with higher grafting densities leading to stronger reductions in mobility. Understanding the effect of this and other parameters will allow for better control and design of electrochemical systems.

#### 2.2.2 Separation: Extending the Functionality of Membranes

Membranes separate molecules based on how easily these molecules pass through them.^[^ ^13^, ^14^ ^]^ This permeability depends strongly on the size distribution of the pores and the charge of the membrane. Hence, control over the pore size distribution and charge can tune the permeability of the membrane, especially if one decreases the size of the largest pores in the system. Often selectivity is based on size or charge (or a combination of both).

**Figure 4:**
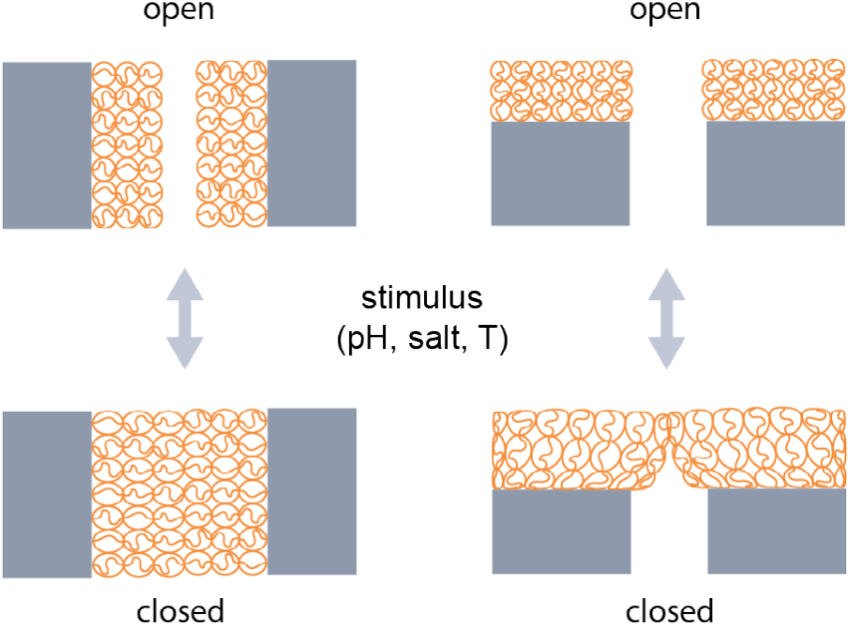
Overview of polyelectrolyte brushes in membrane applications. Brushes can be synthesized inside the pores (left) or on top of the membrane (right).

Some synthetic strategies have been devised that can create membranes with polyelectrolyte brushes in the pores (**Figure** **4**). While introducing polyelectrolyte brushes into pores can be done as a post‐fabrication step, such brush‐coated membranes can be more readily prepared via self‐assembly methods. Zhang et al. synthesized brush‐coated membrane layer with a thickness of 500 nm through the self‐assembly of a triblock copolymer of polyisoprene‐*b*‐polystyrene‐*b*‐poly(acrylic acid) with volume fractions of 0.25: 0.42: 0.33, respectively.^[^ ^132^ ^]^ The self‐assembled structure forms pores coated with PAA chains that can change the pore size by collapsing and swelling through external stimuli. For instance, in 10 mM MgCl_2_ the brushes collapse leading to a larger cut‐off radius compared to deionized water and 10 mM NaCl. Alternatively, one can create brush‐coated pores through the self‐assembly of brush‐coated nanoparticles. Zhang et al. deposited PAA‐coated polystyrene nanoparticles with chain lengths ranging from 25 to 72 nm onto a porous support.^[^ ^133^ ^]^ The resulting structure could then be used to separate nanoparticles based on size by varying the pH of the medium.

Another approach is to functionalize porous membranes directly with polyelectrolyte brushes.^[^ ^134^, ^135^, ^136^ ^]^ In these systems, it is difficult to characterize whether the polymers are inside the pores or merely on top of the substrate. Nevertheless, this modification alters the properties of membranes as demonstrated by Porter et al. who cross‐linked grafted poly[2‐(methacryloyloxy)ethyl trimethylammonium iodide] and PMAA‐co‐HEMA (poly(methacrylic acid‐*co*‐2‐hydroxyethyl methacrylate)) on cellulose supports.^[^ ^137^ ^]^ The functionalization reduced the water permeability and the salt rejection of such membranes became salt‐concentration dependent. Similarly, Khor et al. functionalized a membrane with PAA grown on carbon nanotubes and showed that the resulting membrane had a performance that depended on the applied voltage.^[^ ^138^ ^]^

Finally, one can even use brushes for separations without membranes. Che et al. grew PMETAC brushes on reduced graphite oxide sheets.^[^ ^139^ ^]^ They could extract heavy metal ions from aqueous solution using this method.

Responsive membranes based on polyelectrolyte brushes mainly exploit a change in brush height. A thicker brush can clog or block pores, limiting molecular transport through these pores. For instance, numerical models revealed that weak polyelectrolyte brushes can influence flow through nanochannels.^[^ ^140^ ^]^ Examples from the literature show that salt can be used to modulate molecular transport.^[^ ^141^ ^]^ However, it is not difficult to imagine that height changes resulting from other stimuli such as changes in pH, solvent, electric field, and temperature could result in similar responsive behavior as long as the thickness of the brush can be varied. Design parameters^[^ ^33^, ^34^, ^35^ ^]^ such as grafting density, chain length, charge fraction, and chain length dispersity can play important roles as these provide handles to tune brush height.

#### 2.2.3 Protein Adsorption: Controlling Fouling

The interaction between polyelectrolyte brushes and foulants such as proteins is complicated and many physicochemical processes contribute to this interaction.^[^ ^10^, ^41^, ^142^, ^143^, ^144^ ^]^ Besides charge‐based interactions, one of the driving forces of protein adsorption is counterion release.^[^ ^142^ ^]^ Proteins have an inhomogeneous surface charge with patches of positive and negative charge. On these patches, counterions are more confined than in solution. When these counterions are replaced by a polyelectrolyte chain or other multivalent ions, the confined ions are released and entropy of the system increases. This effect allows proteins to bind to brushes at low salt concentrations even though the net charge of the protein has the same sign as the brush.^[^ ^145^ ^]^ At high salt concentrations, the proteins are released as the charge interactions are screened by the ions in the medium. These effects have been described in more detail in theoretical studies.^[^ ^146^, ^147^ ^]^ The release of ions is influenced by not only by the external concentration of electrolyte, but also by the type; fundamental work on the effect of salt on brushes has revealed that brushes can display significant ion‐specific effects (as discussed in Section 3.1.1).

**Figure 5:**
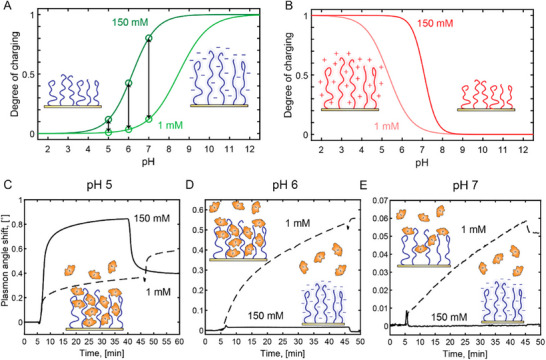
The protonation of weak polyelectrolyte brushes (PMAA–polyacid, PDEA (poly(2‐(diethylamino)ethyl methacrylate))–polybase) depends on both the pH and the ionic strength of the surrounding solution, which affects the fouling by proteins (BSA). Reproduced under terms of the CC‐BY license.^[^ ^11^ ^]^ Copyright 2020, The Authors, published by American Chemical Society.

**Figure 6:**
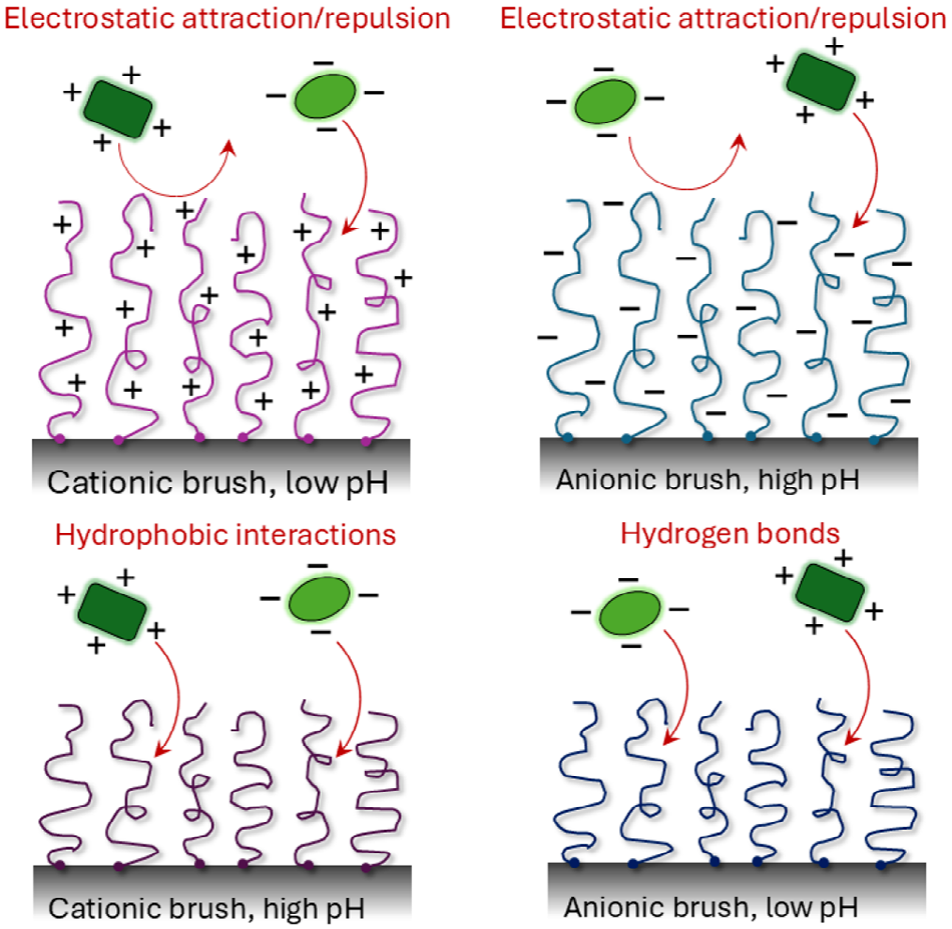
Proteins interact with polyelectrolyte brushes through various mechanism, including electrostatic attraction for ionized brushes, but also hydrophobic interactions and hydrogen bonds at pH where the brushes are not charged. At physiological salt, polyelectrolyte brushes are anti‐fouling only when the protein and brush experience a strong enough electrostatic repulsion. For anionic brushes, this requires that the pH is significantly higher than both the pKa and the protein pI, while for cationic brushes the pH must be much lower than both pKa and pI.

Another explanation can be found for proteins that bind to weak polyelectrolyte brushes. Dense weak brushes experience significant shifts in their pKa values as a result of a locally high concentration of acidic or basic groups, leading to an electrostatically driven change in the acid‐base dissociation equilibrium (see also Section 3.2.1). This change in apparent pKa may lead to an overestimation of the net charge on the polymer, especially for low salt concentrations (see **Figure** **5**).^[^ ^11^ ^]^ If the proteins have an affinity for the neutral brushes, it may be actually this intrinsic affinity that causes binding, rather than electrostatic interactions.^[^ ^144^, ^148^ ^]^ These different effects are summarized in **Figure** **6**.

In experimental studies, the adsorption of proteins on/in polyelectrolyte brushes is found to follow similar trends: adsorption decreases with increasing salt concentration. You et al. studied the adsorption of γ‐globulin (pI 6.9) and recombinant human lactoferrin (pI 8.2) on thin anionic PSPMA brushes.^[^ ^149^ ^]^ They estimated the adsorption of these proteins on the QCM (quartz crystal microbalance) sensor with the Sauerbrey equation and found that more protein adsorbs with increasing chain length and grafting density. By varying the pH and salt concentration of the protein solutions, they showed that protein adsorption can be modulated through the ion concentration. The effect of ion release is reversible when cycling between different salt concentrations. Han et al. studied the adsorption of BSA (bovine serum albumin, pI 4.7) on cationic PMETAC brushes (0.22 chains/nm^2^, 6.0 nm dry thickness) and lysozyme on anionic PSPMA brushes (properties not reported).^[^ ^104^ ^]^ They found that the adsorption was highly reversible when cycling between 0.1 mM and 1.0 M NaCl solutions with adsorption changes of several µg cm^−2^, which is an order of magnitude larger than BSA monolayers on flat substrates.^[^ ^150^ ^]^

The pH of the solution also affects protein adsorption. Wang et al. studied the adsorption of BSA on spherical polybasic PAEMA (poly(2‐aminoethyl methacrylate)) brushes and found a maximum in the adsorption at a pH of 6.1 when they studied a pH‐range from 5.0 to 9.0 with X‐ray scattering on spherical brushes.^[^ ^151^ ^]^ In this study, increasing the salt concentration from 7 to 47 mM did not affect the adsorption significantly. Koenig et al. studied the adsorption of glucose oxidase in polyacidic PAA brushes using spectroscopic ellipsometry and observed maximum adsorption around pH 5.^[^ ^152^ ^]^

The fouling‐resistant behavior of polyelectrolyte brushes also influences their anti‐microbial character. Mixed brushes of poly(*N*‐hydroxyethyl acrylamide) and PMETAC showed excellent anti‐fouling and bactericidal properties against various types of bacteria.^[^ ^153^ ^]^ Similar bactericidal effects were observed in poly(cationic liquid) brushes, where cyclic brushes seemed to be even more bactericidal than their linear counterparts.^[^ ^154^ ^]^ This bactericidal characteristic is also beneficial in membrane applications. In fact, poly(acrylic acid) brushes can be used to mitigate the accumulation of biomass on membranes^[^ ^155^ ^]^ and on materials used in medical equipment.^[^ ^93^ ^]^

The interaction between polyelectrolytes and proteins is highly complex and even though some important aspects have been elucidated, much remains unknown. If we consider electrostatic interactions, one may be able to maximize protein adsorption by optimizing grafting density, charge fraction, or chain length in the brush. Especially the charge fraction can be tuned via external conditions such as pH. Additionally, in cases where adsorption takes place due to counterion release, one should design systems that maximize the entropic gain of released counterions or water near hydrophobic moieties.

#### 2.2.4 Wetting: Brushes Interacting with Liquids

Polyelectrolyte brushes change the interaction of a substrate with liquids.^[^ ^156^ ^]^ In fact, one of the first tests to confirm the successful synthesis of a polymer brush is a contact angle measurement. Brushes not only change the interaction with liquids, this interaction can also change depending on the environmental conditions. Azzaroni et al. demonstrated this using counterion exchange in PMETAC brushes.^[^ ^52^ ^]^ When the chloride counterion was exchanged for different counterions, the water contact angle could be changed from 35° for Cl^−^ to 19° for PO_4_^3−^ and to 79° for ClO_4_^−^. These wetting changes were reversible when the brushes were rinsed with an excess of chloride solution. Wettability can also be changed by electric fields. Sénéchal et al. studied the wetting behavior of self‐assembled brushes of PS‐b‐PAA (polystyrene‐*b*‐poly(acrylic acid)) brushes under a variety of bias potentials where the counterelectrode was placed inside the droplet.^[^ ^157^ ^]^ They found that wetting changed at pH 4 and at biases more negative than ‐500 mV, but only in the presence of electrolyte (2 mM KCl). Spruijt et al. synthesized a PMETAC brush and showed that the water contact angle could be changed depending on the oxidation state of ferricyanide counterions.^[^ ^158^ ^]^

Another interesting interaction with water occurs at low temperatures. The temperature at which heterogeneous ice nucleation takes place on PMETAC and PSPMA brushes depends on the counterion of these brushes.^[^ ^159^ ^]^ Kosmotropic counterions give a lower heterogeneous nucleation temperature than more chaotropic ions in both types of brushes.

Brushes provide a tool to influence the wetting behavior of surfaces.^[^ ^160^ ^]^ Control over the wettability of surfaces may find uses in applications ranging from anti‐fogging,^[^ ^161^ ^]^ and self‐cleaning windows to microfluidic devices.^[^ ^162^, ^163^ ^]^ These wettability changes combined with the presence of specific ions are also partially responsible for the reversibly switchable bactericidal properties of polyelectrolyte brushes.^[^ ^164^, ^165^, ^166^, ^167^, ^168^ ^]^ Additionally, wettability changes might be exploited in molecular separations.^[^ ^34^, ^169^ ^]^ If the affinity toward a minority compound can be tuned, one might be able to use this effect for molecular extractions.

#### 2.2.5 Transducing Molecular Signals for Optical or Electrical Sensing

The stimulus‐response of polyelectrolyte brushes leads to changes in the structure and properties of the brush. These changes can be sensed and brushes can therefore be a transduction mechanism for sensors. For instance, one can measure the optical response when a PNIPAM‐*co*‐AA brush is coated with a thin gold layer, and this response can be linked to changes in the swelling of the brush.^[^ ^170^ ^]^ In maleimide brushes modified with autofluorescent moieties, conformational transitions induced by changes in pH could be directly observed by its autofluorescence.^[^ ^171^ ^]^ Alternatively, Christau et al. related the water content in PDMAEMA brushes to the relative humidity and showed that the composition in swollen brushes is independent of the (dry) brush height.^[^ ^172^ ^]^ The absorbed water leads to swelling, so the brush becomes a humidity sensor if the swelling can be measured. These experiments are in line with previous neutron scattering experiments on PDMAEMA brushes.^[^ ^46^ ^]^

Another system uses PMETAC brushes to immobilize silver nanoparticles. The addition of the nanoparticles turns the PMETAC brush into a stimulus‐responsive system. At pH 5, this system could be used as a very sensitive SERS (surface enhanced Raman spectroscopy) active surface where 4‐ATP at concentrations as low as 10^−8^ M could be detected.^[^ ^173^ ^]^ Wu et al. showed that composite brushes containing PDMAEMA can also be used for the detection of heavy metal ions such as Cu^2+^ and Cd^2+^.^[^ ^174^ ^]^

Changing brush configurations can also be detected via electrical means. For instance, Klinghammer et al. grafted PAA brushes onto a silicon nanowire field‐effect transistor.^[^ ^175^ ^]^ With the resulting device, they could monitor the changes in the conformation of the brushes as a result of pH and ionic strength. Additionally, they showed that the device could facilitate or prevent the adhesion of biomolecules depending on the ionization state of the brush and protein. Another brush system that can be used as a sensor consists of a poly(ionic liquid) brush with ferrocene moieties, which can differentiate between buffer solutions with and without 0.1 mM tyrosine.^[^ ^176^ ^]^

Polyelectrolyte brushes as a transducing mechanism show promise. From earlier work, we know that vapors can adsorb in polymer brushes^[^ ^177^, ^178^, ^179^, ^180^ ^]^ and the absorption can be sensed optically with ellipsometry and mechanically with a quartz‐crystal microbalance.^[^ ^181^, ^182^ ^]^ Similar sensing mechanisms are available to sense proteins and other biomolecules adsorbing from solutions.^[^ ^183^, ^184^, ^185^ ^]^ The desired parameters for these transducer brushes depend on the application. For some applications, sparse, long brushes may be best as they can swell the most while for other such sparse brushes would lead to system deterioration as a result of fouling.

### 2.3 Introducing Electrical and Energetic Functionality

Polyelectrolyte brushes can be used in a variety of electrical and energy applications^[^ ^16^, ^186^ ^]^. Energy applications that use polymer brushes have been summarized recently.^[^ ^15^ ^]^ This excellent review covers topics ranging from the use of polymer brushes in proton exchange membranes, composites for lithium batteries, anodes for lithium batteries, and organic radical batteries to oxygen and hydrogen evolution reactions, high performance supercapacitors, hydrogen production, and photovoltaic devices. In the remainder of this section, we focus on electrical and energetic functionality that were not covered in detail in this review.

Polyelectrolyte brushes are especially important in energy applications that are based on brush‐functionalized nanochannels, where they affect molecular transport by influencing the interaction between the fluid and the channel surface.^[^ ^187^ ^]^ Transport is mainly affected by the energetic interaction of the molecule with the wall material. Depending on the interaction, transport can take place with or without molecular slip at the fluid‐wall interface. This behavior is often characterized using the slip length: The distance from the wall to where the flow velocity is (or extrapolates to) zero. This slip length is an important parameter in nanochannel flow and is influenced by the operation conditions, surface wettability, surface roughness, and liquid viscosity.^[^ ^187^ ^]^ The transport of ions through nanochannels is affected by surface charges and the resulting double layer around this surface charge, similar to capillary electrophoresis. Additionally, not all ions are equally mobile due to size and hydration differences. These combined effects allow one to generate an electrically driven flow by applying an axial bias across the channel. For a detailed description of such transport phenomena, we refer the reader to a recent review by Nazari et al.^[^ ^187^ ^]^

#### 2.3.1 Energy Generation in Brush‐Functionalized Nanochannels

Consider a nanochannel that connects two reservoirs. If the free energy of one reservoir differs from the other, this results in a driving force for transport through the nanochannel. The free energy difference can be due to an imbalance of temperature, concentration, or electrostatic potential. Typically, a system equilibrates these differences through the transport of ions, which leads to a streaming current and streaming potential over the nanochannel. This potential and current can be harvested in the form of electrical energy.^[^ ^188^, ^189^, ^190^ ^]^ Polyelectrolyte brushes grafted inside nanochannels affect the transport through nanochannels. First, brushes affect the slip length of the channel as they act as a lubricating layer that changes the friction between the wall and the liquid. Second, brushes displace the onset of the electric double layer away from the wall. And third, brushes change the ion distribution and ionic composition inside the channel.

Different types of brushes have been studied in detail using mean‐field theories, including end‐charged brushes^[^ ^191^, ^192^ ^]^ and brushes with chargeable moieties along the backbone.^[^ ^193^, ^194^, ^195^, ^196^, ^197^ ^]^ For all of these systems, ion flows were quantified for free energy gradients induced by concentration, thermal, and pressure differences between both reservoirs. These streaming currents were shown to depend significantly on changes in environmental conditions such as pH and ionic strength. Slightly more detailed studies using molecular theory and atomistic simulations have revealed additional interesting features of ionic transport through nanochannels. For instance, with atomistic simulations, Pial et al. found evidence of an overscreening effect in brushes that is affected by large axial electric fields, causing a reversal in the direction of the ionic current as the axial field strength is increased^[^ ^198^ ^]^ as was previously predicted theoretically.^[^ ^199^ ^]^ Sadeghi et al. studied the ionic transport through nanochannels with molecular theory as a function of pH and ionic strength and revealed a interesting interplay between both system parameters.^[^ ^200^ ^]^ Perez Sirkin et al. studied the effect of axial electric fields on the structure of polyelectrolyte brushes in nanochannels and found a transition between collapsed‐to‐the‐center and collapsed‐to‐the‐wall states.^[^ ^201^ ^]^ Finally, Sadeghi et al. studied the implications of ionic currents through nanochannels and found that the induced field is significantly stronger in brush‐coated nanochannels than in bare nanochannels.^[^ ^202^ ^]^ Such detail provide promising indications that these coated nanochannels form interesting systems for future energy applications.

#### 2.3.2 Ion Current Rectification with Ionic Diodes

The flow of ions through nanochannels is quite sensitive to geometry and chemical conditions when the typical size of the channel approaches the Debye length‐scale.^[^ ^203^, ^204^ ^]^ Under these conditions, the ionic resistance can become asymmetrical, leading to a preferred direction for ionic current. In essence, this asymmetric system creates a diode that can rectify ionic currents. Here the important aspect is asymmetry in the overlap of the double layers on both ends of the nanochannel. The asymmetry can be purely geometric,^[^ ^205^ ^]^ but it can also be induced by polyelectrolyte brushes. When a polyelectrolyte brush is swollen under specific pH conditions on one side of the channel, and not on the other side of the channel, then this generates an asymmetry that can create a current rectification effect.^[^ ^206^, ^207^ ^]^ The key parameter here is the Debye length relative to the minimum pore diameter. Most work has been done on well‐defined small‐scale systems, where for practical applications this needs to be reproduced over larger surface areas (many channels in parallel) and this will present a significant challenge.

**Figure 7:**
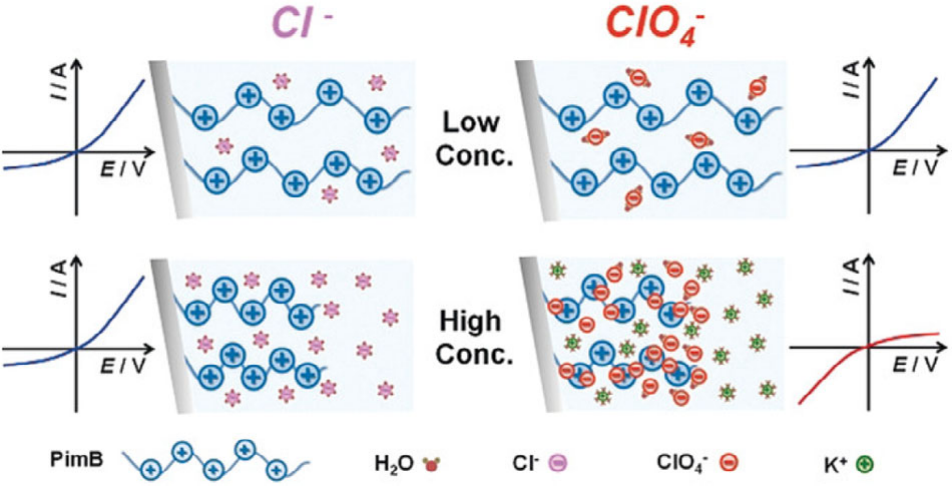
Schematic illustration of brush‐coated ionic diodes with a conical shape. The current rectification behavior changes for high concentrations of ClO_4_^−^ compared to low concentrations and compared to Cl^−^ solutions. Reproduced with permission. [208] Copyright 2018, Wiley.

Changing the charge state of the polymer can tune the effective pore size and the double layer structure simultaneously. For instance, pH and voltage responsive DNA brush coatings can be applied in nanopores to create a pH‐responsive ion gate.^[^ ^209^ ^]^ Similarly, one can coat the diodes with PAA to create pH‐gated nanochannels.^[^ ^210^ ^]^ An intrinsic feature of the gate is the (degree of) overlap of the double layers at the pore opening. A way to influence the surface charge on this tip is through counterion localization: When more counterions localize, the surface potential and subsequent double layer change (see **Figure** **7**). This can be achieved by counterion exchange and has been demonstrated using a variety of chaotropic ions.^[^ ^208^ ^]^

While the difference in double‐layer overlap can be achieved via a conical shape of the nanochannel, the asymmetry can also be created by an asymmetric response of the bushes in cylindrical nanochannels. If the brushes are pH‐swollen on one end, and collapsed at the other end, the double layer overlap varies along the length of the channel, giving a current rectification. Depending on the axially applied voltage, a current inversion can be observed.^[^ ^206^ ^]^ As stated previously, the main properties affecting the diode behavior relate to the brush height (influencing the effective pore diameter).

## 3 Stimulus‐Response Behavior

**Figure 8:**
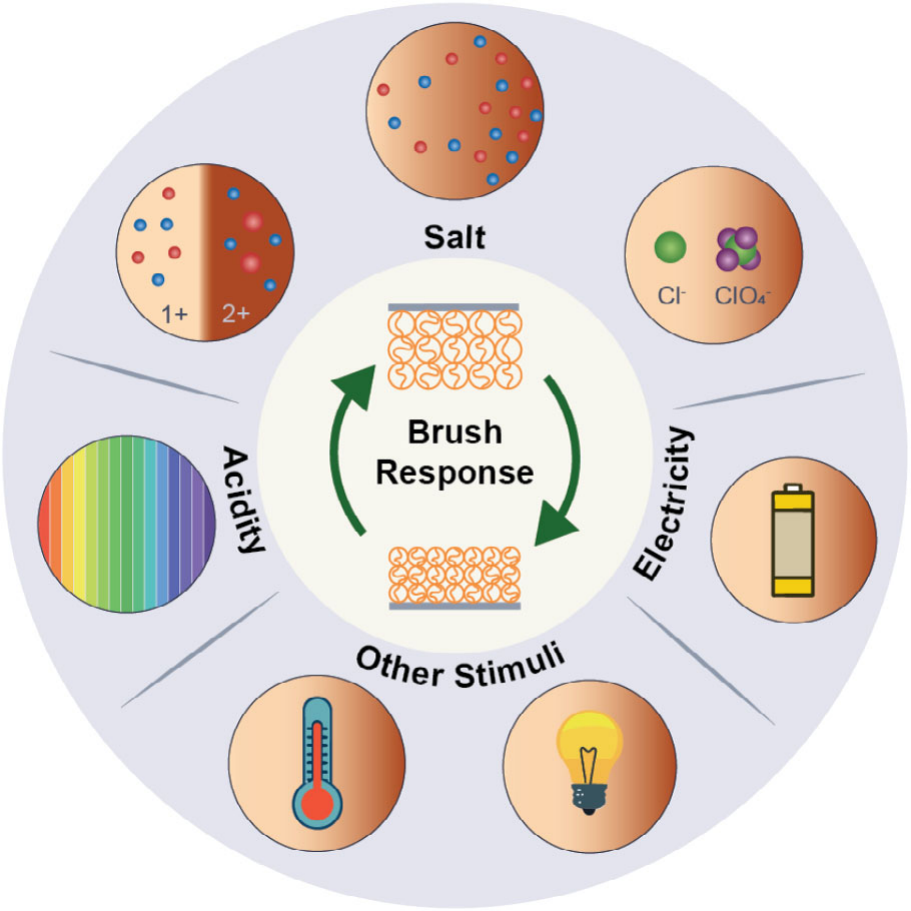
Overview of different stimuli that have been used to change the properties of responsive polyelectrolyte brushes. These include stimuli introduced via the medium (salt–3.1, acidity–3.2) and externally (electricity–3.3, temperature–3.4.1, light–3.4.1).

In Section 2, we reviewed various applications and use cases of polyelectrolyte brushes and the effect of structural changes in these brushes. However, multiple environmental factors can affect the structure and properties of polyelectrolyte brushes as well as the entities they interact with. How can one influence the structure of a brush? Which factors influence the behavior of a brush? As illustrated in **Figure** **8**, we review the effect of salt (Section 3.1), pH (Section 3.2), and electric fields (Section 3.3) on the structure and properties of polyelectrolyte brushes (Figure 8). Additionally, we briefly mention more general stimuli that can induce responses in polyelectrolyte brushes but also in their uncharged counterparts (Section 3.4) such as temperature, solvent quality and light.

### 3.1 The Effect of Salts and Ions

#### 3.1.1 Monovalent Ions

**Figure 9:**
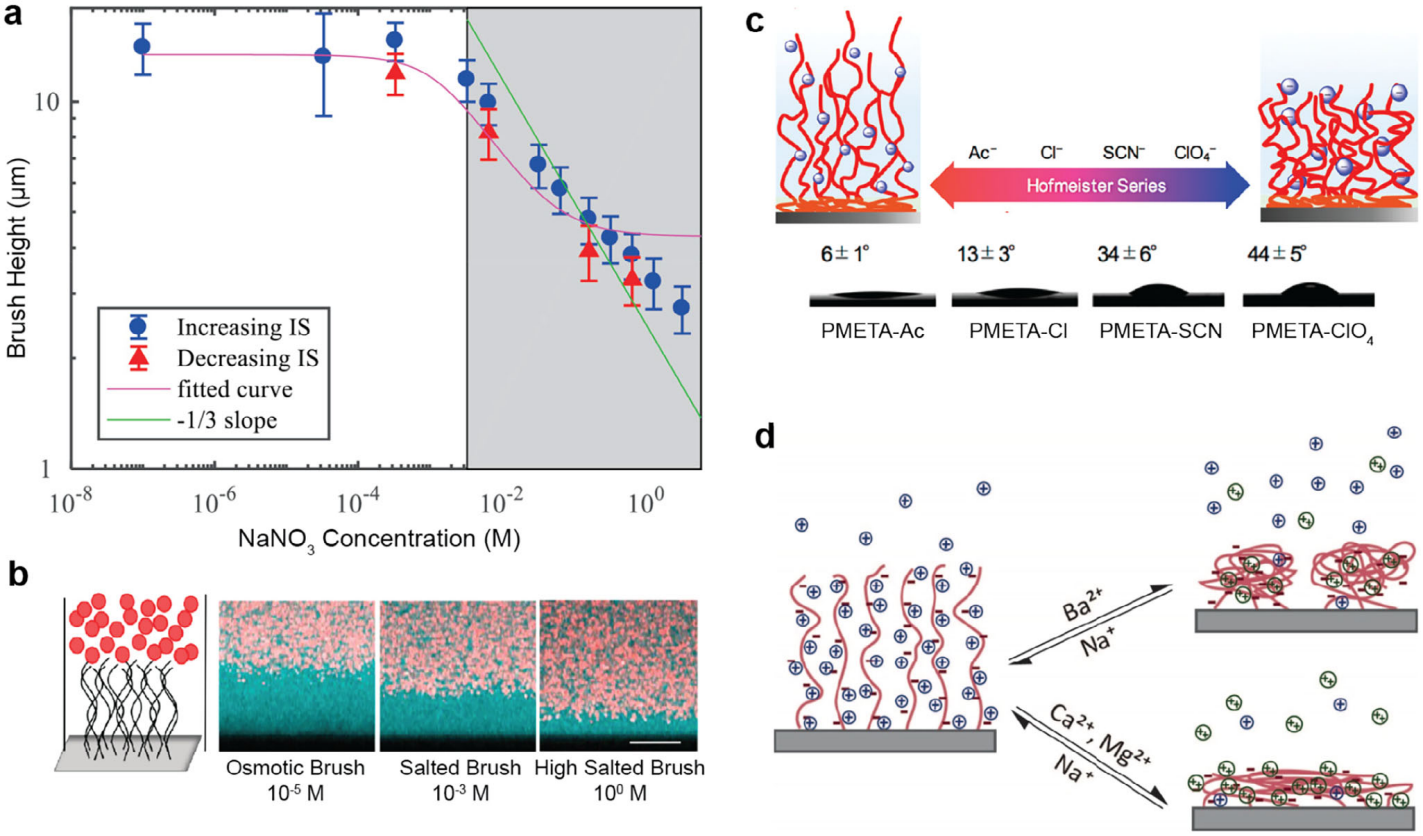
Effect of salt on the properties of polyelectrolyte brushes. a) The brush height as a function of the salt concentration with the osmotic regime at low concentrations and the salted regime with a characteristic scaling exponent near ‐1/3 at high concentrations. Adapted with permission.^[^ ^212^ ^]^ Copyright 2019, American Chemical Society. b) As the salt concentration increases, brushes go from osmotically swollen to a salt‐induced collapsed state. Adapted with permission.^[^ ^212^ ^]^ Copyright 2019, American Chemical Society. c) The wettability of polyelectrolyte brush surfaces changes with counterions following the Hofmeister series. Adapted with permission.^[^ ^218^ ^]^ Copyright 2018, American Chemical Society. d) Multivalent ions induce a collapse of polyelectrolyte brushes with collapse feature depending on the chemistry of the multivalent ions. Reproduced with permission.^[^ ^219^ ^]^ Copyright 2019, American Chemical Society.

Polyelectrolyte brushes carry charges along their chains and these charges are accompanied by oppositely charged counterions. Pincus^[^ ^72^ ^]^ first theorized the structure of polyelectrolyte brushes and identified three length scales that characterize a polyelectrolyte brush: one related to the concentration of charged monomers in the brush (the charge neutralization length, ξ), one related to the ionic strength of the solution (the Debye screening length, κ^−1^), and one related to the brush itself (the brush height, *H*). Based on these lengths, he identified three different regimes: the osmotic regime (ξ ⩽ *H* ≪ κ^−1^) where counterions are confined to the brush and create an osmotic pressure that leads to a swollen conformation; the Pincus regime (ξ > *H* and κ^−1^ > *H*) where the counterions form a diffuse layer into the solution that screens the charge on the brush; and the salted regime (κ^−1^ < ξ ⩽ *H*) where the osmotic pressure difference is negligible and charge interactions inside the brush are additionally screened. Depending on the regime, the response to salt has different theoretical scaling behavior (see **Figure** **9**a,b). In the osmotic regime, the brush height is independent of the salt concentration since it is dominated by the osmotic pressure of the counterions. In the salted regime, the brush height scales as *c* ^−1/3^ as charged interactions are screened. Since Pincus' theory does not account for all effects in polyelectrolyte brushes, the scaling exponent in the salted regime has been found to be lower than the theoretical value.^[^ ^211^, ^212^, ^213^, ^214^, ^215^ ^]^ Besides the brush height, salt also affects other properties of a polyelectrolyte brush such as its structure,^[^ ^216^ ^]^ interfacial properties,^[^ ^217^ ^]^ water structure,^[^ ^69^ ^]^ and (counter)ion mobility.^[^ ^27^, ^69^ ^]^

In dense brushes of strong polyelectrolytes (for instance PSPMA, PSS, or PMETAC), the polymer and its counterions can form dipole aggregates that cause a more compact brush structure. Ions in the surrounding medium weaken these aggregates, leading to a swelling of these brushes when the salt concentration is increased. When around 10 and 100 mM salt is added to highly charged brushes like PSPMA, PSS, or PMETAC, the height of these brushes can increase compared to their salt‐free height.^[^ ^220^, ^221^ ^]^ The kinetics of the disruption of these dipole aggregates with increasing salt concentration are faster than their reformation under decreasing salt concentration.^[^ ^222^ ^]^

Not only the concentration of counterions matters, the chemistry of the counterion plays a significant role as well. When PMETAC brushes were imaged with tapping mode atomic force microscopy, a surprising difference between brushes with chloride and perchlorate counterions was found: Brushes with chloride counterions were soft and deformed if the normal force of the AFM cantilever was increased whereas those with perchlorate counterions were stiff and could not be deformed even at higher AFM normal forces.^[^ ^223^ ^]^ Such ion‐specific effects were also observed with quartz crystal microbalance,^[^ ^224^ ^]^ total internal reflection microscopy,^[^ ^225^ ^]^ and contact angle measurements.^[^ ^218^ ^]^ Similar ion‐specific effects have later been observed in various systems including PDMAEMA,^[^ ^226^ ^]^ PDPA (poly[(2‐diisopropylamino)ethyl methacrylate]),^[^ ^227^ ^]^ and PDMMAA‐*b*‐PMAA (poly(*N*,*N*′‐dimethylmethylacrylamide‐*b*‐poly(methacrylic acid)) brushes.^[^ ^228^ ^]^

Ion‐specific effects often follow the Hofmeister series. In this series, the ions are arranged depending on their tendency to help dissolve proteins (chaotropes) or precipitate them (kosmotropes). If one orders ions by increasing tendency to collapse polyelectrolyte brushes, one finds a similar sequence.^[^ ^71^, ^229^, ^230^ ^]^ For instance, when the chloride ions in PMETAC are exchanged with other ions, the hydrophilicity (measured by water contact angle) gradually increases in the following order: perchlorate > thiocyanate > iodide > bromide > chloride > phosphate,^[^ ^52^ ^]^ as illustrated in Figure 9c. The origin of these ion‐specific effects have not yet been elucidated completely but they have been linked to the hydration energy of the ions^[^ ^231^ ^]^ and the binding strength between the counterions and the brush.^[^ ^71^, ^232^ ^]^ The binding strength between brush and counterion might be predicted based on quantum‐mechanical evaluation of these ions since the electrostatic potential maximum of ions correlates to the interaction energy.^[^ ^233^ ^]^ The hydration and binding effects are sometimes described in combination as ion pairing;^[^ ^234^ ^]^ in general, ions that pair stronger with the brush contribute less to the osmotic pressure and therefore result in a more compact brush. Additionally, the mobility of water molecules that solvate ions could affect ion interactions with the brush.^[^ ^235^, ^236^ ^]^ This also means that ion‐specific effects are susceptible to brush design parameters such as the grafting density.^[^ ^237^ ^]^ Despite our incomplete understanding regarding the origin of ion‐specific effects, the consequences of ion‐specific effects–such as ion partitioning and brush structure–could be satisfactorily described through an effective interaction between the ion and polyelectrolyte brushes in self‐consistent field calculations.^[^ ^65^, ^238^, ^239^, ^240^ ^]^ In fact, these effects can even create a partitioning of similarly charged ions,^[^ ^241^ ^]^ which is especially relevant in water purification applications.

Properties of a polyelectrolyte brush vary with the counterion, which makes these counterions a design parameter. The introduction of charged molecules as counterions can provide polyelectrolyte brushes with new functionality. For instance, NaPSS brushes can become thermoresponsive through the introduction of phosphonium ions.^[^ ^242^ ^]^ Alternatively, the introduction of sodium dodecyl sulphate as counterions could lead to an inversion in the zeta‐potential of PDMAEMA brushes at pH 9.^[^ ^243^ ^]^

Finally, we note that not all ions induce changes in the brush. The inclusion of very bulky ions can be prevented through steric effects. As a result, the ionic strength of the solution can be modified without affecting the brush.^[^ ^230^ ^]^

#### 3.1.2 Multivalent Ions

Multivalent ions strongly interact with polyelectrolyte brushes and even trace amounts can significantly impact the structure and properties of brushes.^[^ ^244^, ^245^ ^]^ The collapse in solutions of trivalent ions is most drastic and has been studied in a variety of systems, including bidisperse brushes,^[^ ^246^ ^]^ four‐arm polyelectrolyte brushes,^[^ ^247^ ^]^ ring architectures,^[^ ^248^ ^]^ and brushes grafted to a variety of colloids.^[^ ^113^, ^248^, ^249^, ^250^, ^251^ ^]^ An example of the effect of multivalent ions on polyelectrolyte brushes is the appearance of fractal‐like patterns on DNA‐functionalized microchips when they are exposed to trivalent spermidine^3+^.^[^ ^252^ ^]^ When these DNA brushes were exposed to 65 µM spermidine^3+^, the brushes collapsed into dendritic, hairlike structures. This striking behavior was subsequently studied using coarse‐grained molecular dynamics simulations^[^ ^253^, ^254^, ^255^ ^]^ and it was found that such trivalent ions can induce laterally inhomogeneous structures in polyelectrolyte brushes. In fact, if the rigidity of the grafted chain is taken into account, simulations of polyelectrolyte brushes in trivalent salt can recover this dendrite formation.^[^ ^256^ ^]^ Interestingly, the ions in these dendritic structure are rather mobile, which may be conducive for future applications. It is also interesting that only some divalent ions (Ba^2+^) show similar structure formation, while other ions lead to a more homogeneous collapse (Ca^2+^, Mg^2+^) (Figure 9d).^[^ ^211^, ^219^ ^]^

The multivalent ion‐induced collapse of a polyelectrolyte brush resembles the collapse of brushes in poor solvents^[^ ^257^ ^]^ but shows some distinct differences. First, the brushes that experience a trivalent‐ion induced collapse contain more short‐ranged structure as a result of the highly‐charged nature (and therefore stronger screening) of the counterions.^[^ ^258^ ^]^ Second, the brushes collapse into aggregates that appear to be smaller than under solvent‐induced collapse^[^ ^258^ ^]^ or form laterally inhomogeneous structures whereas those do not form for equivalent brushes in solvent‐induced collapse. Brettmann et al.^[^ ^259^ ^]^ constructed a theory that compares the free energy of pinned micelle structures with pinned cylindrical structures. The model recovered brush heights that are more in line with experimental values presented in this study than their previous mean‐field model.^[^ ^260^ ^]^ When the adhesion between two opposing PSS brushes was measured using a surface force apparatus, the brushes adhered to each other upon retraction under solvent‐collapsed conditions, while the brushes repelled each other under ion‐induced collapse conditions.^[^ ^261^ ^]^

The effect of multivalent ions can be described using two contributions: a chemistry specific contribution and a pure multivalency effect. For trivalent ions, the latter effect usually dominates while for divalent ions the former comes into play.^[^ ^219^ ^]^ For instance, PSS brushes collapse at mM concentrations of barium, while collapse as a result of magnesium ions only starts at concentrations two orders of magnitude higher.^[^ ^211^ ^]^ The type of counterion also affects the hydrogen‐bond structure of water in the brush.^[^ ^68^ ^]^

Finally, multivalent ions often show an additional swelling transition at high salt concentrations.^[^ ^262^, ^263^ ^]^ As the PSS brush goes from no added salt conditions to a solution of 1 to 10 mM, the dipole‐dipole associations in the brush break up and the brush swells. Then with increasing salt concentrations, the brushes enter an osmotic regime (for monovalent ions) or form laterally‐inhomogeneous structures as a result of salt‐aggregation (for multivalent ions) leading to a collapse of the brush. Finally, at even higher concentrations on the order of 0.1 M, the brushes with multivalent ions as well as cesium experience a reswelling.

### 3.2 The Effect of Acid

#### 3.2.1 The pH‐Response of Weak Polyelectrolyte Brushes

**Figure 10:**
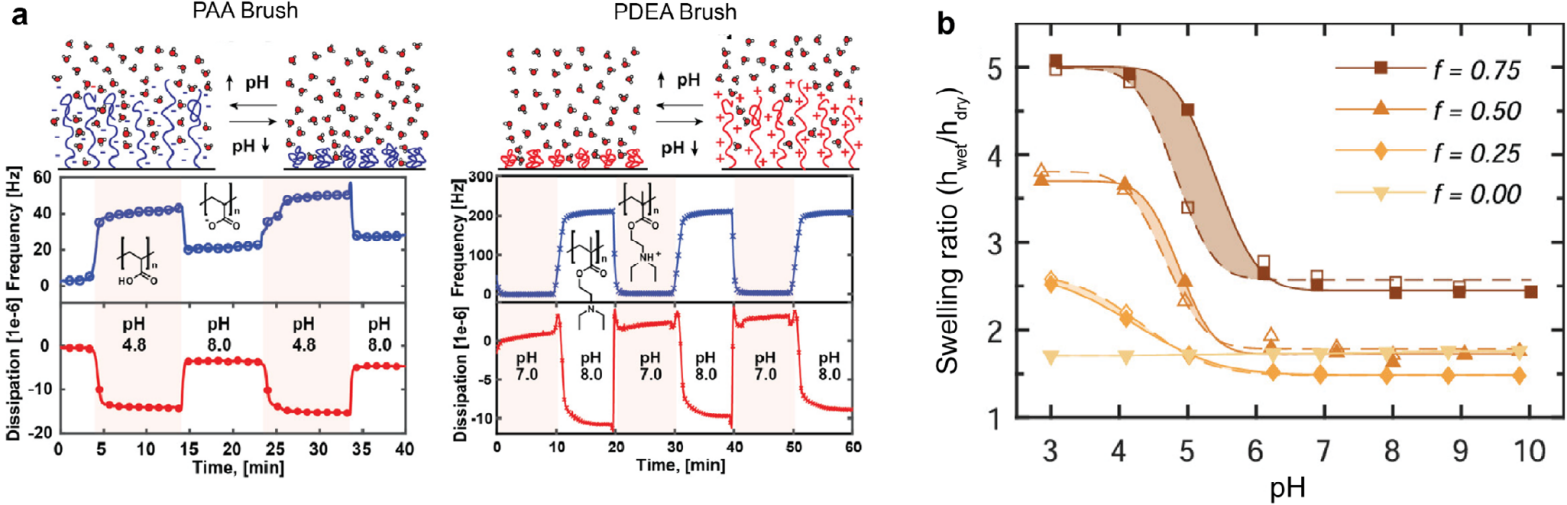
pH‐response of polyelectrolyte brushes. a) Frequency and dissipation shift upon switching the pH of acidic PAA and basic PDEA brushes. Reproduced under terms of the CC‐BY license.^[^ ^48^ ^]^ Copyright 2018, The Authors, published by American Chemical Society. b) Brushes with different fractions of pH‐responsive monomers show different pH‐response with increasing hysteresis with increasing fraction of pH‐responsive monomers. Adapted with permission.^[^ ^264^ ^]^ Copyright 2023, American Chemical Society.

Weak polyelectrolytes contain pH‐responsive acidic or basic moieties that (de)protonate depending on the local pH. For instance, commonly used examples of weak polyelectrolyte brushes contain carboxylic acid moieties (for example PAA or PMAA) or amine moieties (for example PDMAEMA). When moieties change their protonation state, this introduces or removes charges in brushes leading to a change in brush height.^[^ ^265^, ^266^ ^]^ **Figure** **10** **a** illustrates such a response in a typical QCM‐D experiment. The reported frequency changes when the protonation state changes: a lower frequency indicates a higher wet mass on the sensor as a result of the brush swelling. Such a change can also be reported via Förster resonance energy transfer (FRET) when the chains are labeled with FRET acceptors and donors.^[^ ^267^ ^]^ While these moieties have well‐defined pKa values as monomers in solution, the apparent pKa of brushes can differ by several pH units from this value.^[^ ^48^, ^268^ ^]^ Beyer et al.^[^ ^28^ ^]^ found that the shift in apparent pKa (roughly logarithmically with salt concentration) in polyelectrolyte brushes results from a combination of the Donnan effect (the partitioning of ions, specifically protons, in regions with fixed charges) at high grafting density combined with the polyelectrolyte effect (the change in apparent pKa due to electrostatic repulsion between acidic or basic moieties) at low grafting density. The polyelectrolyte effect is nearly independent of salt concentration, but depends on the grafting density. In fact, the authors claim the apparent pKa might be used to measure the brush grafting density.^[^ ^48^ ^]^ Additionally, in solutions with divalent ions, the pH‐response showed a distinct two‐stage swelling as a consequence of charge regulation and ion partitioning.^[^ ^269^ ^]^

Hollingsworth et al.^[^ ^47^ ^]^ studied the pH response of PAA brushes with a non‐grafted PEI (poly(ethylene imine)) layer on top. They found that the pH response shows a hysteresis for minutes for long chains at low grafting density, for short time scales at intermediate grafting densities and chain lengths, and no hysteresis for short dense brushes. Bajgiran et al.^[^ ^264^, ^270^ ^]^ studied the effect of the fraction of chargeable sites on the pH‐response of weak polyelectrolyte brushes (PDMAEMA‐co‐HEA, poly[2‐(dimethylamino)ethyl methacrylate‐*co*‐2‐hydroxyethyl acrylate]). They found that all charge‐containing brushes are pH‐responsive, and the transition pH (apparent pKa) shifts to more basic values with increasing fraction of ionizable monomers (Figure 10b). At intermediate pH ranges, all systems show hysteresis in swelling but there is no hysteresis in the zeta‐potential measurements. The authors therefore hypothesize a height‐dependent pH‐response where the top reacts quicker than the lower parts of the brush.

The apparent pKa shift is further influenced by the ionic strength of the solution. With increasing salt concentration, the apparent pKa of self‐assembled spherical brushes of PDMAEMA‐b‐PSS moves closer to the pKa of the pure monomer.^[^ ^271^ ^]^ Additionally, the magnitude of pH‐response is also affected by salt; for instance, the pH‐induced structural change of PDMAEMA brushes weakens as the salt concentration increases from 10 to 100 mM because the osmotic pressure at low pH is limited in this brush.^[^ ^272^ ^]^ Besides the ionic strength, the ion type affects the pH response as well. Willott et al.^[^ ^273^ ^]^ studied the effect of different counterions on PDPA brushes using AFM measurements with a silica‐colloid probe. The adhesion depended on the counterion: thiocyanate always formed adhesive interactions with the colloid; acetate only showed adhesion at low salt concentration (<0.13 mM), while nitrate showed adhesion at low (0.04 mM) and high (100 mM) salt concentrations, but not at intermediate concentrations (0.13 – 10 mM).

#### 3.2.2 The pH‐Response of Strong Polyelectrolyte Brushes

The pH‐response of weak polyelectrolyte brushes is caused by a change in protonation (and thus charge) of the residues in the polymer, which is affected by pH, local composition, and the formation of local structures. While the degree of ionization is constant in strong polyelectrolyte brushes, these brushes can still show a pH‐responsive behavior. As the solution pH changes, the ratio of OH^−^ and H_3_O^+^ changes and this affects the hydrogen bonding network of the water in these brushes. This composition change causes a pH‐response in the strong polyelectrolytes such as PSPMA,^[^ ^274^ ^]^ PMETAC,^[^ ^274^ ^]^, and PSS^[^ ^275^ ^]^ since the hydration of the polyelectrolyte is affected. Molecular theory shows that OH^−^ and salt‐ion mediated bridging can lead to a pH response in the polyelectrolyte brush.^[^ ^276^ ^]^

### 3.3 Electricity

When polyelectrolyte brushes are placed in an electric field, a variety of interactions come into play. On the one hand, electric fields exert forces on charges, dipoles, and polarizable groups in the brush. On the other hand, these fields can also affect the system by inducing redox reactions at electrode interfaces, which often consume or produce protons, thus affecting the dissociation equilibria of pH‐responsive moieties. These effects often occur concurrently, which creates a complex interplay between various direct and indirect responses. To unravel this complexity, electricity as a stimulus has been studied with experiments,^[^ ^118^, ^158^, ^277^, ^278^, ^279^, ^280^, ^281^, ^282^ ^]^ with theory and computations,^[^ ^60^, ^283^, ^284^, ^285^, ^286^, ^287^, ^288^ ^]^ and with simulations,^[^ ^289^, ^290^, ^291^, ^292^, ^293^, ^294^, ^295^, ^296^ ^]^ and increased understanding gradually paves the way toward applications.

#### 3.3.1 Theoretical Exploration of the Electroresponse

**Figure 11:**
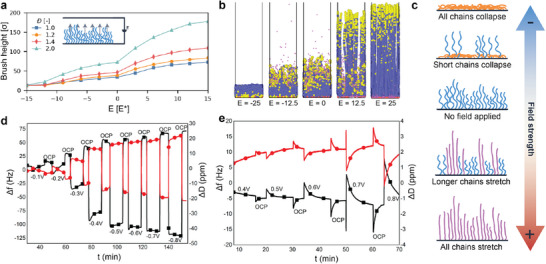
Electroresponsive properties of polyelectrolyte brushes. a) Coarse‐grained simulations reveal that dispersity increases the electroresponse of disperse brushes under salt‐free conditions. Adapted under terms of the CC‐BY license.^[^ ^33^ ^]^ Copyright 2025, The Authors, published by American Chemical Society. b) Snapshots of coarse‐grained simulations on the electroresponse of gradient polyelectrolyte brushes; yellow beads–charged monomers, blue beads–neutral monomers. Adapted under terms of the CC‐BY license.^[^ ^35^ ^]^ Copyright 2024, The Authors, published by American Chemical Society. c) Disperse brushes show a chain‐length dependent response to electric stimuli. Adapted under terms of the CC‐BY license.^[^ ^33^ ^]^ Copyright 2025, The Authors, published by American Chemical Society. d) QCM‐D measurements of a poly(acrylic acid) brush with various negative electrode potentials; black–frequency, red–dissipation. Adapted with permission.^[^ ^280^ ^]^ Copyright 2015, American Chemical Society. e) QCM‐D measurements of a poly(acrylic acid) brush with various positive electrode potentials; black–frequency, red–dissipation. Adapted with permission.^[^ ^280^ ^]^ Copyright 2015, American Chemical Society.

In repulsive fields, the charges of the polyions in the brush experience a force away from the grafting plane. These electric forces can lead to a deformation of the brush (**Figure** **11**a,b): The height of a brush increases in repulsive fields.^[^ ^284^, ^295^, ^297^ ^]^ The effect of the electric field on the brush height is stronger for sparsely‐grafted polyelectrolyte brushes with a low charge density than for denser brushes with a higher charge density. This charge density effect was later also found in self‐consistent field theory calculations.^[^ ^286^ ^]^ Stretching in electric fields occurs via a bimodal transition: As the strength of the field increases and a critical field strength is reached, an increasing number of chains stretch out, leading to unstretched, partially‐stretched, and fully‐stretched structures.^[^ ^292^, ^295^ ^]^ This effect can also be seen in brushes with a charge gradient where the free ends of the polymer are charged (Figure 11b). In attractive fields, the charges experience a force toward the grafting plane. Under these conditions, brushes collapse via a similar bimodal transition where increasingly many chains collapse as the field strength increases.^[^ ^284^, ^287^, ^291^ ^]^ In fact, if one considers a brush consisting of only the polymers in the unperturbed subpopulation, the height and profile of this brush is equivalent to the height of the collapsed brush.^[^ ^284^, ^291^ ^]^ Interestingly, for disperse brushes these subpopulations are stratified by chain length: shorter chains collapse first, while longer chains stretch first (Figure 11c).^[^ ^33^ ^]^ The field‐induced transition is bimodal because it is ‘cheaper’ in terms of free energy to deform individual chains by a lot than all chains by a little.^[^ ^287^ ^]^

Theoretical work on the electroresponse of polyelectrolyte brushes revealed that grafting density plays a key role in the response characteristics of these brushes, even when the grafted polyion chains are not branched and monodisperse. However, polyelectrolyte brushes can be designed from a wide variety of systems including curved substrates,^[^ ^298^ ^]^ linear polyions with varying chain stiffness,^[^ ^293^ ^]^ linear polyions with a composition gradient along the polymer backbone,^[^ ^34^, ^35^ ^]^ block copolymers with neutral and charged blocks,^[^ ^299^ ^]^ branched polyions with three arms^[^ ^133^ ^]^ or four arms,^[^ ^300^ ^]^ and brushes with polyions with different chain lengths.^[^ ^33^, ^285^, ^290^ ^]^ These different architectures subtly affect the electroresponse of the brushes. These studies show the importance of macromolecular design in responsive systems.

Most theoretical studies have been performed in the absence of electrolyte ions. As discussed in Section 3.1, salt drastically affects the properties of polyelectrolyte brushes and the surrounding medium will contain electrolytes in most practical use cases, and most probably as a complex mixture of various ions. Ions can screen the electric field through the formation of double layers, which may reduce the electroresponse of polyelectrolyte brushes and reduce the number of polyelectrolyte chains that restructure in applied fields.^[^ ^286^, ^287^, ^293^ ^]^ In fact, brushes retain their electroresponse in solutions with a low concentration of electrolyte (typically around 10 mM), while at higher salt concentrations they lose their responsive character.^[^ ^296^ ^]^ This loss in response seems to correlate with the transition from a brush in the osmotic regime to one in the salted regime. To exploit the electroresponse of polyelectrolyte brushes in a wide range of applications, it will be crucial to understand how counterions of different valence and types influence the response characteristics.

For weak polyelectrolyte brushes, changes in ionization limit the response in brush thickness relative to the applied voltage.^[^ ^284^ ^]^ Surface charges influence the local pKa of the acidic or basic moieties similar to the polyelectrolyte effect (see Section 3.2.1) and hence the ionization varies with distance from the grafting plane, leading to a non‐monotonous height change.^[^ ^284^ ^]^ Additionally, ions are generally more mobile than large polyions, which introduces multiple time scales in these systems. In simulations and experiments, such different time scales have been observed where the system shows a quick initial response and a slower relaxation to its equilibrium state.^[^ ^280^, ^293^ ^]^

So far, the electric fields have all been applied perpendicular to the grafting plane. In some applications, one needs to apply a potential in the axial direction. Pial et al. studied the effect of an axial field on polyelectrolytes using all‐atom molecular dynamics simulations.^[^ ^301^ ^]^ The response of the polyions under such conditions depends on the charge density on the pendant group: polyions with less charge density in the pendant group (for example PSS) tilt when an axial field is applied as the field acts on this charge, while polyions with a higher charge density in the pendant group (for example PAA or PMAA) bend as the field causes the counterion cloud around the pendant group to polarize and effectively counteract the effect of the electric field.

#### 3.3.2 Experiments on Electroresponsive Brushes

The available literature on experiments of the electroresponse of polyelectrolyte brushes is sparse and most studies are done using weak polyelectrolyte brushes. In these systems, one not only probes the electroresponse of the brush, but also the effect of potential‐induced changes in ionization, creating a complex interplay of effects.

The direct electroresponse of PDMAEMA brushes under salt‐free conditions was studied using ellipsometry and neutron reflectometry by Weir et al.^[^ ^282^ ^]^ The neutron reflectometry data indicates that the brushes stretch via the bimodal response as predicted by simulations. However the critical applied bias is large with 3.0 V and chains started to get ripped from the substrate at 5.0 V with an electrode separation of 3 mm. With negative biases, collapse did not exceed the experimental uncertainty. Unfortunately, the pH of the system was not controlled, so the observations can be affected by unobserved electrode reactions.

Since weak polyelectrolyte brushes can change their ionization state, this can affect their response depending on the local pH of the system. Sénéchal et al. studied the electroresponse of PAA and PDMAEMA using E‐QCMD.^[^ ^279^ ^]^ They uncovered three different response regimes: i) uncharged weak polyelectrolyte brushes, ii) strongly charged weak polyelectrolyte brushes, and iii) brushes near their pKa. The first regime hardly responds to external fields. The second regime shows prompt response to electric fields, but the response is partially irreversible and shows hysteresis. More salt reduces the response of the brush if it is charged (regime ii), but enhances the response of the system if the brush is uncharged (regime i). The third regime shows a typical two‐phase response: first a quick swelling/collapse followed by a gradual relaxation. This two‐phase response was also observed in PAA brushes under slightly alkaline conditions (pH 8).^[^ ^280^ ^]^ Surprisingly, QCM experiments showed a decrease in frequency when a negative potential was applied to the brush‐coated electrode (Figure 11d), indicating an increase in the wet mass and swelling of the brush. On the other hand, the frequency increased when a positive potential was applied (Figure 11e), indicating a decrease in the wet mass and a collapse of the brush. In both cases, the height shows a quick and large change immediately after the potential is first applied and later the brush height settles at a smaller change. The collapse when a negative potential is applied can be explained by oxygen reduction reactions.^[^ ^10^ ^]^ Balzer et al.^[^ ^283^ ^]^ provided an additional explanation for the inconsistency in the electroresponse of weak polyelectrolyte brushes. They posed that–when a small potential is applied–ions from the solution enter the brush to screen these charges. Without this screening, the surface charge would deprotonate the polyacid leading to swelling.^[^ ^284^ ^]^ Additionally, the adhesion between PAA brushes^[^ ^277^ ^]^ as well as the water contact angle^[^ ^157^ ^]^ decrease as a negative bias is applied.

Besides these strong and weak polyelectrolyte brushes, brushes can also be made electroresponsive through the inclusion of redox‐active moieties. Ferrocene‐containing polymers have been grafted to glassy carbon, indium tin oxide and gold electrodes.^[^ ^176^ ^]^ The wetting behavior of the resulting system differed between the different oxidation states with ferrocene in its reduced form leading to the highest contact angles. Ferrocene‐containing polymers have also been grafted from polystyrene particles, making them redox‐active.^[^ ^302^ ^]^ Other redox‐active moieties that can be used are 2,2,6,6‐tetramethylpiperidin‐1‐oxyl‐4‐yl (TEMPO) groups which contain a stable radical and can oxidize to an oxoammonium cation.^[^ ^303^, ^304^, ^305^ ^]^ These systems may find uses in energy storage.

### 3.4 Other Stimuli

Some stimuli can induce responsiveness in both charged and uncharged brushes. While these stimuli are not exclusive to polyelectrolyte brushes, they still provide a valuable method to control the properties of these brushes. These general stimuli include temperature (Section 3.4.1), solvent quality (Section 3.4.2), and light (Section 3.4.3).

#### 3.4.1 Temperature

Temperature is a common stimulus in stimulus‐responsive coatings. There are two strategies to create a temperature response. First, one can use the intrinsic thermoresponsiveness of the polymer or induce thermoresponsiveness via external means. A polymer with an intrinsic thermoresponsiveness is PDMAEMA. This polymer has a variable LCST that varies with pH^[^ ^306^ ^]^ and maintains this responsiveness even when it is grafted at high densities (1.4 nm^−2^) in a copolymer with photoresponsive^[^ ^307^ ^]^ or photocrosslinkable^[^ ^308^ ^]^ monomers. Second, one can introduce thermoresponsiveness via counterion exchange in a PSS brush^[^ ^242^ ^]^ or by copolymerizing charged and thermorespo nsive monomers (for example P(NIPAM‐*co*‐MAA (poly(*N*‐isopropylacrylamide‐*co*‐methacrylic acid)).^[^ ^309^ ^]^ These strategies are complementary and can be combined in PDMAEMA brushes when the chloride counterion is exchanged with ferricyanide, changing the lower critical solution temperature (LCST) behavior of the brush into a upper critical solution temperature (UCST) behavior.^[^ ^310^ ^]^ Finally, the thermoresponse of brushes is affected by other external factors, for instance adsorbed gold nanoparticles can stifle the thermoresponse of PDMAEMA brushes.^[^ ^307^ ^]^ We note that thermoresponsive systems can show excellent reversibility, however, it is important to note that increasing temperature can have significant negative effects in biological media and protein solutions.

#### 3.4.2 Solvent Quality

The response of polyelectrolyte brushes (as well as all polymers) depends on the solvent quality (sometimes expressed via the excluded volume in models^[^ ^193^ ^]^) of the surrounding medium. Water is an exceptional solvent with a very high dielectric constant, so replacing it with other solvents will increase the Bjerrum length and lead to less ion dissociation and non‐electrostatic interactions gain relative importance over osmotic and electrostatic interactions.^[^ ^311^ ^]^ The viscoelastic structure of PSS brushes changes when methanol is added to the surrounding medium.^[^ ^262^ ^]^ Similarly, hyaluronan brushes collapse drastically upon an increase of the ethanol fraction in surrounding medium with an especially sharp collapse between 70 % and 80 % ethanol (v/v).^[^ ^212^ ^]^ Differences in swelling were also observed in atomistic simulations when PMETAC and PDMAEMA brushes were studied in water and cyclohexane.^[^ ^226^ ^]^ Hence, changes in solvent can also be exploited as a stimulus.

#### 3.4.3 Light

Polyelectrolyte brushes are not natively light‐responsive. However, one can introduce light‐responsive character using similar approaches as we saw previously for thermoresponsive brushes. A first approach consists of exchanging counterions with azobenzene containing ions that have UV/Vis induced cis‐trans isomerization.^[^ ^312^, ^313^, ^314^ ^]^ This approach is conceptually straightforward, yet changes in the system upon light‐induced isomerization are often not very pronounced. Nevertheless, isomerization can create mechanical tension that can be exploited to create surface patterns based on interferometry patterns.^[^ ^314^ ^]^ A second approach consists of creating polyelectrolyte brushes with light‐responsive comonomers.^[^ ^99^, ^308^, ^315^ ^]^ Several types of comonomers have been studied in literature, including photo‐cross‐linkable^[^ ^308^ ^]^ and azobenzene containing^[^ ^99^, ^315^ ^]^ comonomers in a PDMAEMA brush. These systems have enabled some degree of control over the swelling of brushes^[^ ^308^ ^]^ and ion transport^[^ ^99^, ^315^ ^]^ within them.

## 4 Design Considerations

### 4.1 Chemistry and Materials Considerations

The previous sections established that many stimuli can be used to change the properties of polyelectrolyte brushes, especially when it comes to the height of the brush. The brush height, and consequently the polymer density, are the easiest property to monitor and it directly influences many of the functions discussed in Section 2, such as the ability of the brush to create barriers or control adsorption of biomolecules. The stimulus‐response of these systems has been demonstrated in various types of brushes containing one or more types of monomers. **Table** **2** provides an overview of monomers for which responsive properties have been reported. This table serves as a starting point for the design of a brush for specific applications. Note that weak polyelectrolytes are generally more responsive to pH than strong polyelectrolytes and that ionized polyelectrolytes are more responsive to salt than acidic and basic brushes in their neutral state.

**Table 2:** Monomers for polyelectrolyte brushes reported in selected recent references.

Monomer	Full name	Remarks	Synthesis method^a)^
*Weak cationic / basic*			
PDMAEMA	poly[2‐(dimethylamino)ethyl methacrylate]	Thermoresponsive	SI‐PET‐RAFT^[^ ^98^, ^161^, ^267^ ^]^
			SI‐RAFT^[^ ^164^ ^]^
			SI‐ATRP^[^ ^119^, ^137^, ^232^, ^311^, ^316^, ^317^ ^]^
			SI‐ARGET‐ATRP^[^ ^227^, ^318^ ^]^
			ATRP^[^ ^36^ ^]^
PDMAEA	poly[2‐(dimethylamino)ethyl acrylate]		SI‐Cu(0)‐ATRP^[^ ^264^, ^270^ ^]^
PDEA	poly[2‐(diethylamino)ethyl methacrylate]		SI‐ARGET‐ATRP^[^ ^11^, ^148^, ^227^ ^]^
			SI‐ATRP^[^ ^119^ ^]^
PDPA	poly(2‐diisopropylamino)ethyl methacrylate)		SI‐ARGET‐ATRP^[^ ^65^, ^227^, ^240^, ^273^ ^]^
P2VP	poly(2‐vinylpyridine)		N/A^[^ ^22^, ^23^ ^]^
*Strong cationic*			
PMETAC	poly{2‐[(methacryloyloxy)ethyl]‐trimethylammonium chloride}		SI‐ATRP^[^ ^16^, ^87^, ^104^, ^126^, ^218^, ^229^, ^231^, ^234^, ^319^ ^]^
			SI‐ARGET‐ATRP^[^ ^49^, ^320^ ^]^
			SI‐PET‐RAFT^[^ ^161^, ^321^ ^]^
			SI‐Cu(0)‐ATRP^[^ ^322^ ^]^
PMEPTAC	poly{3‐(methacryloylamino)propyl]‐trimethylammoniumchloride}		SI‐PET‐RAFT^[^ ^161^ ^]^
PBIM	poly[2‐(1‐butylimidazolium‐3‐yl)ethyl methacrylate]		SI‐ATRP^[^ ^237^ ^]^
PVBIM	poly(1‐vinyl‐3‐butylimidazolium)		SI‐ATRP^[^ ^208^ ^]^
*Weak anionic/ acidic*			
PAA^b)^	poly(acrylic acid)		RAFT^[^ ^36^ ^]^
			ATRP^[^ ^278^ ^]^
			N/A^[^ ^47^ ^]^
			SI‐ARGET‐ATRP^[^ ^11^, ^144^, ^148^ ^]^
			SI‐ATRP^[^ ^323^ ^]^
PMAA	poly(methacrylic acid)		SI‐PET‐RAFT^[^ ^161^ ^]^
			SI‐ATRP^[^ ^98^, ^165^, ^313^ ^]^
			SI‐ARGET‐ATRP^[^ ^11^, ^144^ ^]^
			photograft polymerization^[^ ^166^ ^]^
*Strong anionic*			
PSPMA	poly(3‐sulfopropyl methacrylate)	Probably crosslinked	UV‐photopolymerization^[^ ^324^ ^]^
			SI‐ATRP^[^ ^87^, ^104^, ^120^, ^121^, ^149^, ^167^, ^168^, ^234^, ^245^ ^]^
			SI‐ARGET‐ATRP^[^ ^49^, ^325^ ^]^
			SI‐PET‐RAFT^[^ ^161^, ^321^ ^]^
PSS	poly(styrenesulfonate)		SI‐ATRP^[^ ^51^, ^217^, ^219^, ^221^, ^232^, ^245^, ^261^ ^]^

^a)^ ATRP–atom transfer radical polymerization; RAFT–reversible‐addition chain‐transfer polymerization; SI–surface initiated; PET–photoelectron transfer; SET–single electron transfer; N/A–polymerization method not reported or commercial polymer used.

^b)^ PAA is sometimes created via postmodification of poly[(*tert*‐butyl)acrylate].

The selection of an initial system consists of two main aspects: selecting an appropriate chemistry and choosing the right physical parameters. For the chemistry, one should consider whether the system needs to be pH‐responsive and whether the brush charge should be positive or negative. For pH‐responsive systems, Table 2 presents options for weak cationic/ basic polyelectrolytes for positive brush charges and weak anionic/acidic polyelectrolytes for negative brush charges. For systems without inherent pH‐response, the table provides options for strong cationic (positive charge) and anionic (negative charge) polyelectrolytes. If the application requires additional responses (for instance thermoresponsiveness or UV/Vis response), additional monomers can be selected from the table and a copolymer could be grown.

One can consider more complicated brush architectures when multi‐responsiveness is desired. One option is the inclusion of two different types of polymers in the same brush to create a mixed polymer brush.^[^ ^326^ ^]^ Such mixed brushes can respond to similar stimuli as single‐polymer brushes, but with an additional option to introduce multi‐stimulus responsiveness through the second polymer, for instance by including PNIPAM. Similarly, one can consider brushes consisting of multiple monomers that each have specific stimulus‐response, for instance a copolymer of the thermoresponsive 2‐(2‐methoxyethoxy)ethyl methacrylate and the pH‐responsive PDEA where the ratio between these monomers determines the dominant response modality.^[^ ^327^ ^]^

Next, one should consider the physical parameters such as grafting density and chain length. Experimental control over these parameters can be challenging; in silico experiments can provide complementary insights to laboratory experiments in this case. For instance, with coarse‐grained molecular dynamics simulations it was shown that grafting density, charge fraction and distribution along the polymer chain as well as the chain length distribution all affect the electroresponse of polyelectrolyte brushes.^[^ ^33^, ^34^, ^35^ ^]^ Similarly, self‐consistent field calculations have revealed various trends between brush design parameters and the brush response.^[^ ^239^, ^240^, ^284^, ^309^, ^328^ ^]^ With increasing computational power, even atomistically resolved simulations may become available to study the response behavior of polyelectrolyte brushes.^[^ ^70^, ^329^, ^330^ ^]^ While the effect of brush parameters on the response of the brush can be explored using in silico experiments, these methods can only make predictions as good as the model that was used. In real‐world applications, effects can be influenced by unforeseen and difficult to control additional effects such as contaminants or a limit to temperature control. Therefore, it is important to validate the predicted results with experimental observations.

### 4.2 Indirect Responses to Stimuli

The response of a polyelectrolyte brush does not always directly follow from the stimulus that is imposed. For instance, the application of electric fields can both directly affect the properties of a brush by acting on the charges in the grafted polymer, but it can also act on the counterions and indirectly affect the brush via the osmotic pressure of these additional counterions.^[^ ^118^ ^]^ Additionally, redox reactions can locally change the pH of the solution which can affect the charge state of weak polyelectrolyte brushes. For instance, by applying strong voltages on an electrode that has a weak polyelectrolyte brush, one can create a coupling between this applied field, the pH response wave, and the height of the brush.^[^ ^331^ ^]^ In a similar manner, Joule heating as a result of a current or IR absorption may be used to create an indirect thermoresponse due to local heating. These indirect responses may aid in the design of brushes that change properties at specific locations in the system. Alternatively, magnetic heating could be used for brushes grafted to magnetic substrates. Finally, one could also employ gaseous stimuli such as CO_2_ and N_2_ to influence the pH of the medium and in turn the properties of the brush.^[^ ^332^, ^333^ ^]^

### 4.3 Synthetic Considerations

**Figure 12:**
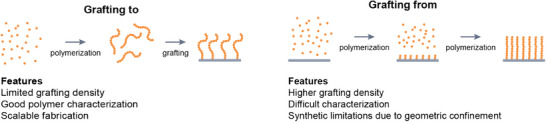
Schematic overview of the two main methods to fabricate polymer brushes and their main features.

There are two main approaches to synthesizing a polymer brush: grafting to and grafting from. These approaches are illustrated in **Figure** **12** and come with their own strong and weak points. In grafting to Ref.[334], one first synthesizes a polymer (or a precursor polymer) with a functional terminal end. Then this polymer is attached to the substrate via a chemical reaction or physical interaction between the substrate and the functional polymer. To graft chains to the substrate, one can ensure the presence of complementary reactive groups on the substrate and the polymer. This method allows for better control and characterization of the grafted polymers, however achieving high grafting densities is difficult, unless special strategies are employed.^[^ ^36^, ^38^ ^]^

Alternatively, one can use a grafting from approach. Here one initiates the polymerization from the substrate. Many different polymerization chemistries have been used to this end (Table 2) with many variations to optimize the procedure for reliability, efficiency, and ease‐of‐use.^[^ ^7^, ^42^, ^43^, ^44^ ^]^. The most commonly used polymerization method for polyelectrolyte brushes is surface‐initiated atom‐transfer radical polymerization (SI‐ATRP), making it a good starting point to synthesize these brushes. Surface‐initiated reversible‐addition chain‐transfer polymerization (SI‐RAFT) can also be used to grow polyelectrolyte brushes, but the charged nature of the monomers complicates the synthesis compared to neutral monomers.^[^ ^161^, ^321^ ^]^ Grafting from generally creates denser brushes, however, determining the grafting density and macromolecular design (for example the monomer and chain length distribution) is extremely challenging, even using state‐of‐the‐art characterization methods.^[^ ^7^, ^44^ ^]^ Both synthetic approaches have been demonstrated on a wide variety of substrates, ranging from well‐defined flat substrates (for example^[^ ^42^ ^]^ silica wafers, mica, gold) to substrates with complicated morphologies (for example membrane supports^[^ ^134^, ^135^ ^]^ or fabric^[^ ^335^ ^]^).

Another aspect of brush preparation relates to post‐synthesis modifications. Although a great variety of properties and responsiveness to environmental stimuli can be achieved by the broad selection of monomers (Table 2) and co‐polymers thereof, some types of functionality requires further modifications.^[^ ^336^ ^]^ This includes in particular conjugation of biomolecules, which may be receptors for affinity‐based capture^[^ ^337^ ^]^ or enzymes for catalytic function.^[^ ^129^ ^]^ Polyelectrolyte brushes based on carboxylic acids are interesting for conjugation by the well‐known EDC/NHS protocol. Similarly, tertiary amine groups in PDMAEMA can be post‐functionalized with various halogen compounds, for instance for anion removal from water.^[^ ^316^ ^]^ Alternatively, proteins can be immobilized by electrostatic interactions given that the pH is in between the pKa of the brush and the pI of the protein.^[^ ^10^, ^144^, ^148^ ^]^

For reliable results, it is imperative to properly characterize the responsive systems during the various stages of the synthesis.^[^ ^338^ ^]^ For grafting to, this includes a characterization of the molecular weight and weight distribution of the polymers (for example using gel permeation chromatography) before the polymer is grafted to the substrate. For grafting from, this includes a validation of each step in the synthesis procedure including the deposition of the anchor, initiator, and polymer.^[^ ^7^ ^]^ These characterizations include methods such as contact angle measurements, surface energy measurements, atomic force microscopy, X‐ray photoelectron spectroscopy, time‐of‐flight secondary ion mass spectroscopy (ToF‐SIMS), single‐molecule force spectroscopy, and infrared or other optical measurements such as ellipsometry.

## 5 Outlook

In this review, we described how polyelectrolyte brushes can perform various functions in applications and how functionality can be influenced by external stimuli. Through a combination of experimental methods and computational models, our understanding of polyelectrolyte brushes and their response characteristics expanded significantly. However, many challenges remain.

One key challenge relates to the complexity that arises when polyelectrolyte brushes are used in applications that rely on interactions of a brush with various aspects of its environment. Nature provides inspiration on how to exploit this complexity. For instance, polyelectrolyte (bottle)brushes play a key role in the lubrication of our joints through interactions with other components in our synovial fluid.^[^ ^339^ ^]^ When combined with water, these glycoprotein brushes create a good lubricant.^[^ ^340^ ^]^ Similarly, the slipperiness of longsnout catfish depends on their muscle tension, indicating that lubrication performance depends on the stiffness of the underlying substrate, an effect that has recently been recreated in a model system.^[^ ^341^ ^]^

Another key challenge in the field is bringing together computational and experimental work. In computational work, one has complete control over the design parameters (grafting density, chain length, monomer distribution, etc.), but one is limited by the amount of detail and computational cost of models. In experiments, it is difficult to control brush synthesis with a high degree of accuracy and reproducibility in the design parameters. These limitations result from inherent experimental challenges related to growing brushes as well as from fundamental limitations in characterizations. To illustrate, the grafting density–a key design parameter–is very challenging to control and characterize accurately and requires assumptions regarding the density of the grafted layer or the dispersity of the grafted chains.^[^ ^338^ ^]^ To bridge the gap between in silico and on silico experiments, advances in brush synthesis and characterization are crucial. On the other hand, simulations can be brought closer to experiments through the implementation of realistic brush characteristics such as chain length dispersity, monomer distributions, and stochastic distributions of chain anchor points. When experimental and modeling approaches can be made to converge, this will establish a relationship between composition, structure, and application, enabling the rational design of polyelectrolyte brushes.

To move the use of polyelectrolyte brushes from research to practical implementation, several key aspects need to be addressed in the synthesis and functioning of these systems. For instance, the polymers in a brush exist in a thermodynamically frustrated state which is a driving force for the detachment of chains^[^ ^342^, ^343^ ^]^ by (hydrolytic) cleavage of their surface bonds.^[^ ^344^ ^]^ Such degrafting of polymer brushes is more pronounced when the brushes are swollen in good solvents and thereby more stretched.^[^ ^322^, ^345^ ^]^ But it can even occur in brushes that are exposed to only trace amounts of water.^[^ ^346^ ^]^ For polyelectrolyte brushes, one has to be extra careful, since the presence of salt^[^ ^347^ ^]^ and increasing the pH can enhance degrafting.^[^ ^317^ ^]^ To combat chain detachment, several strategies have been devised to increase the stability of brushes through different anchor layers, which has been reviewed in ref. [347]. For example, hydrophobic blocks have been used to prevent water from reaching hydrolysis sensitive surface bonds,^[^ ^323^, ^348^, ^349^, ^350^ ^]^ or macromolecular initiators can be synthesized that enhance the stability by their many (covalent^[^ ^351^ ^]^) surface bonds.^[^ ^352^, ^353^, ^354^ ^]^ The challenge will be to scale up these stable anchoring techniques to larger substrates such that they can eventually be employed in for example the medical field or industrial applications.

Most experiments are performed on (very) small substrates, yet for reliable application it is necessary to functionalize surfaces with brushes^[^ ^355^ ^]^ not only in a repeatable and concise way (for instance through parallel brush synthesis on multiple substrates^[^ ^356^ ^]^), but also larger surface areas would need to be treated. Grafting to is a promising method to scale up the surface area of polymer brushes since this separates the production of the coating material from its application. However, grafting to methods are inherently limited in the grafting density that can be achieved. Scalable grafting from methods have been designed to address this limitation, but these come with additional challenges. First, most grafting from methods rely on controlled radical reactions and these are terminated by oxygen. Oxygen tolerant synthesis methods^[^ ^43^, ^45^, ^357^, ^358^, ^359^, ^360^, ^361^ ^]^ are therefore an important step toward the large scale application of these brushes. Second, compared to the amount of coating applied, the volume of reaction medium in conventional laboratory setups is rather substantial (on the order of tens of mL per cm^2^). To coat large surfaces, it is therefore imperative to reduce solvent consumption for instance by reducing the reaction medium to a confined thin film.^[^ ^318^, ^325^, ^362^, ^363^ ^]^ Finally, the polymerization reaction needs to be initiated at the surface, requiring large surface areas to be coated with initiator molecules for instance via a roll‐to‐roll application.^[^ ^364^ ^]^

Simultaneously, the design and fabrication of new technologies requires careful consideration of sustainability. This sustainability should be addressed both in the fabrication, use, and the end‐of‐life of these coatings^[^ ^365^ ^]^ as well as the processes in which they are employed. Recent work has made some progress in several of these aspects. For example, environmentally friendly polymerization reactions have been designed.^[^ ^366^ ^]^ Gradually the range of monomers has been expanded for bio‐degradable monomers^[^ ^367^, ^368^, ^369^, ^370^, ^371^, ^372^ ^]^ in the case of neutral brushes and a similar development can be done in polyelectrolyte brushes. In parallel, research on the recyclability of bulk polymers^[^ ^373^, ^374^, ^375^ ^]^ may also apply to polyelectrolyte brushes.

Despite the opportunities the application of polyelectrolyte brushes may hold, as demonstrated in many model systems, it is important to keep in mind that for practical application, also other aspects need to be kept in mind. For example, when used in the analysis of medical samples, the separation would need to be specific, even if many other components are present, and that is far from trivial. For application in food, similar arguments can be brought forward, and besides, to warrant food safety, food production lines need to be cleaned regularly using high acid and alkaline conditions, to which the polyelectrolyte brushes would need to be resistant. In that sense, very locally changing the pH by electrode reactions could be a way out, and a specific feature of some of the systems described in this review, and that competing technologies would not have. Last but not least, the repeatability of switching behavior is of essence for application in separation processes that are intended to be operated 24/7. For that, also the effect of irreversibility of binding of the target molecule, or any of the expectedly many present in a practical feed solution would need to be investigated. Possibly advanced docking simulations may be instrumental in determining these effects early on.

In this review, we have highlighted the various stimuli that can be used as well as the chemical and physical design parameters in these brushes. The parameter space in these systems is complex while our ability to properly characterize these systems is rather limited. For instance, the grafting density is difficult to determine reliably and so is the chain length dispersity. The use of active machine learning may be a valuable tool in the design of polymer brushes. As input features, one could use synthesis conditions in conjunction with the characterization methods that are available as well as in situ tests. These machine learned models can the guide the experimental efforts and may even help to identify patterns that may not be directly obvious to a human observer.

## Conflict of Interest

The authors declare no conflict of interest.
